# E3 ligase FBXW7 restricts M2-like tumor-associated macrophage polarization by targeting c-Myc

**DOI:** 10.18632/aging.202293

**Published:** 2020-12-01

**Authors:** Lijia Zhong, Yuanyuan Zhang, Mengyao Li, Yinjing Song, Danhui Liu, Xin Yang, Dehua Yang, Hao Qu, Lihua Lai, Qingqing Wang, Zhimin Chen

**Affiliations:** 1 Department of Pulmonology, The Children’s Hospital, Zhejiang University School of Medicine, National Clinical Research Center for Child Health, Hangzhou 310052, China; 2 Sir Run Shaw Hospital, Zhejiang University School of Medicine, Hangzhou 310016, China; 3 Institute of Immunology, Zhejiang University School of Medicine, Hangzhou 310058, China; 4 Department of Orthopedic Surgery, The Second Affiliated Hospital of Zhejiang University School of Medicine, Hangzhou 310009, China

**Keywords:** FBXW7, tumor-associated macrophages, macrophage polarization, c-Myc, ubiquitination

## Abstract

FBXW7 functions as an E3 ubiquitin ligase to mediate oncoprotein degradation via the ubiquitin-proteasome system in cancer cells, effectively inhibiting the growth and survival of tumor cells. However, little is known about the functions of FBXW7 in macrophages and the tumor immune microenvironment. In this study, we find that FBXW7 suppresses M2-like tumor-associated macrophage (TAM) polarization to limit tumor progression. We identified a significant increase in the proportion of M2-like TAMs and aggravated tumor growth in mice with myeloid FBXW7 deficiency by subcutaneous inoculation with Lewis lung carcinoma cells (LLCs). When stimulated with LLCs supernatant *in vitro*, FBXW7-knockout macrophages displayed increased M2 macrophage polarization and enhanced ability of supporting cancer cells growth. In mechanism, we confirmed that FBXW7 inhibited M2-like TAM polarization by mediating c-Myc degradation via the ubiquitin-proteasome system. These findings highlight the role of FBXW7 in M2-like TAM polarization and provide new insights into the potential targets for cancer immunotherapies.

## INTRODUCTION

Macrophages are particularly abundant in the tumor immune microenvironment and their density is associated with poor prognosis of several cancers [1, 2]. Macrophages are highly plastic and are broadly classified into the pro-inflammatory subset (M1) and the immunosuppressive subset (M2) [3]. Most tumor-associated macrophages (TAMs) are polarized into the M2 subtype by specific factors expressed in the tumor microenvironment [4]. M2-like TAMs participate in cancer initiation, development and metastasis by improving the invasive properties of tumor cells, remodeling the invasive extracellular stroma, promoting angiogenesis, and benefiting tumor cells proliferation [5–10]. At the same time, M2-like TAMs mediate immunosuppression by secreting inhibitory cytokines and upregulating the expression of inhibitory receptors [11–14]. However, the detailed mechanisms of macrophage polarization and its relationship with tumorigenesis remain largely unknown. Exploring the regulators of macrophage polarization and targeting the switch that controls the direction of TAM polarization may improve the efficiency of existing cancer therapies and lead to the development of new cancer immunotherapies [15, 16].

Ubiquitination is a common post-translational modification of intracellular proteins that plays a significant role in cell cycle, signal transduction, proliferation, apoptosis, and immune response [17–21]. Ubiquitination occurs through a cascade of enzymatic reactions involving three different classes of enzymes: ubiquitin-activating enzymes (E1), ubiquitin-conjugating enzymes (E2), and ubiquitin-ligating enzymes (E3) [22]. An abnormality in any one of these enzymes can cause many diseases, including cancer [23, 24]. The E3 ubiquitin ligase F-box and WD repeat domain-containing 7 (FBXW7) is a well-known tumor suppressor that mediates degradation of several oncoproteins including c-Myc, cyclin E, and Mcl-1, which play a critical role in regulating the cell cycle, DNA damage and repair, signal transduction, and transcription factor activity in tumor cells [24] [25]. Therefore, FBXW7 suppresses tumor cell survival and proliferation, effectively limiting cancer development. FBXW7-knockout in mouse embryonic fibroblasts decreased E-cadherin expression and induced the occurrence of epithelial-to-mesenchymal transition, which promotes tumor metastasis [26]. *FBXW7* mutations in bone marrow stromal cells (BMSCs) can increase the CCL2 expression, which promotes recruitment of immunosuppressive cells and metastasis [27]. These findings indicate that FBXW7 can affect cancer development through both tumor cells themselves and through the surrounding non-malignant cells. However, the role of FBXW7 in tumor immune microenvironment, particularly in TAM polarization, has not yet been described.

Recent studies suggest that FBXW7 can mediate CCAAT/enhancer-binding protein delta (C/EBPδ) degradation to suppress *Tlr4* expression, attenuating inflammation, and regulating the innate immune response of macrophages to pathogen [28]. Our previous study showed that FBXW7 catalyzes SHP2 degradation via the ubiquitin-proteasome system to stabilize RIG-I to promote IFN-I production in macrophages, which subsequently orchestrates innate immune response against RNA virus infection [29]. Since FBXW7 plays an integral role in macrophage function, we hypothesized that it might regulate macrophage phenotype switching in the tumor microenvironment. Here, we investigated the role of myeloid cell-specific FBXW7-knockout on tumor progression in mice and on M2-like TAMs. We found that myeloid cell-specific FBXW7-deficient (Lysm^+^FBXW7^f/f^) C57BL/6 mice showed exacerbated tumor progression and had a higher proportion of M2-like TAMs in solid tumor tissues after the subcutaneous injection of Lewis lung carcinoma cells (LLCs). FBXW7 inhibited M2 macrophage polarization and repressed the production of tumor-promoting factors with the stimulation of LLC supernatant *in vitro*. In mechanism, FBXW7 catalyzed the ubiquitination and degradation of c-Myc, which restricts M2-like macrophage polarization. Altogether, our findings clarify the previously unrecognized role of FBXW7 in regulating the function of M2-like TAMs, and provide new potential target for anti-cancer therapies.

## RESULTS

### FBXW7 deficiency in myeloid cells promotes tumor progression in an LLC-inoculated lung cancer model

*FBXW7* mutants have been previously identified in various tumor tissues [30]. Mice with FBXW7 conditional depletion in the T cell lineages, bone marrow stromal cells could develop cancer [27]. To determine whether FBXW7 affects tumor progression by regulating the function of innate immune cells in remodeling the tumor immune microenvironment, myeloid cell-specific FBXW7-deficient (Lysm^+^FBXW7^f/f^) C57BL/6 mice were generated. After confirming the efficiency of FBXW7 knockout (Supplementary Figure 1A–1C), cells in the bone marrow and spleen from FBXW7^f/f^ and Lysm^+^FBXW7^f/f^ mice were collected and analyzed by flow cytometry. We observed no significant differences between FBXW7^f/f^ and Lysm^+^FBXW7^f/f^ mice in terms of the percentage of myeloid cells and lymphocytes (Supplementary Figure 1D–1I). Thus, FBXW7 knockout in myeloid cells did not affect the development of myeloid cells and lymphocytes.

**Figure 1:**
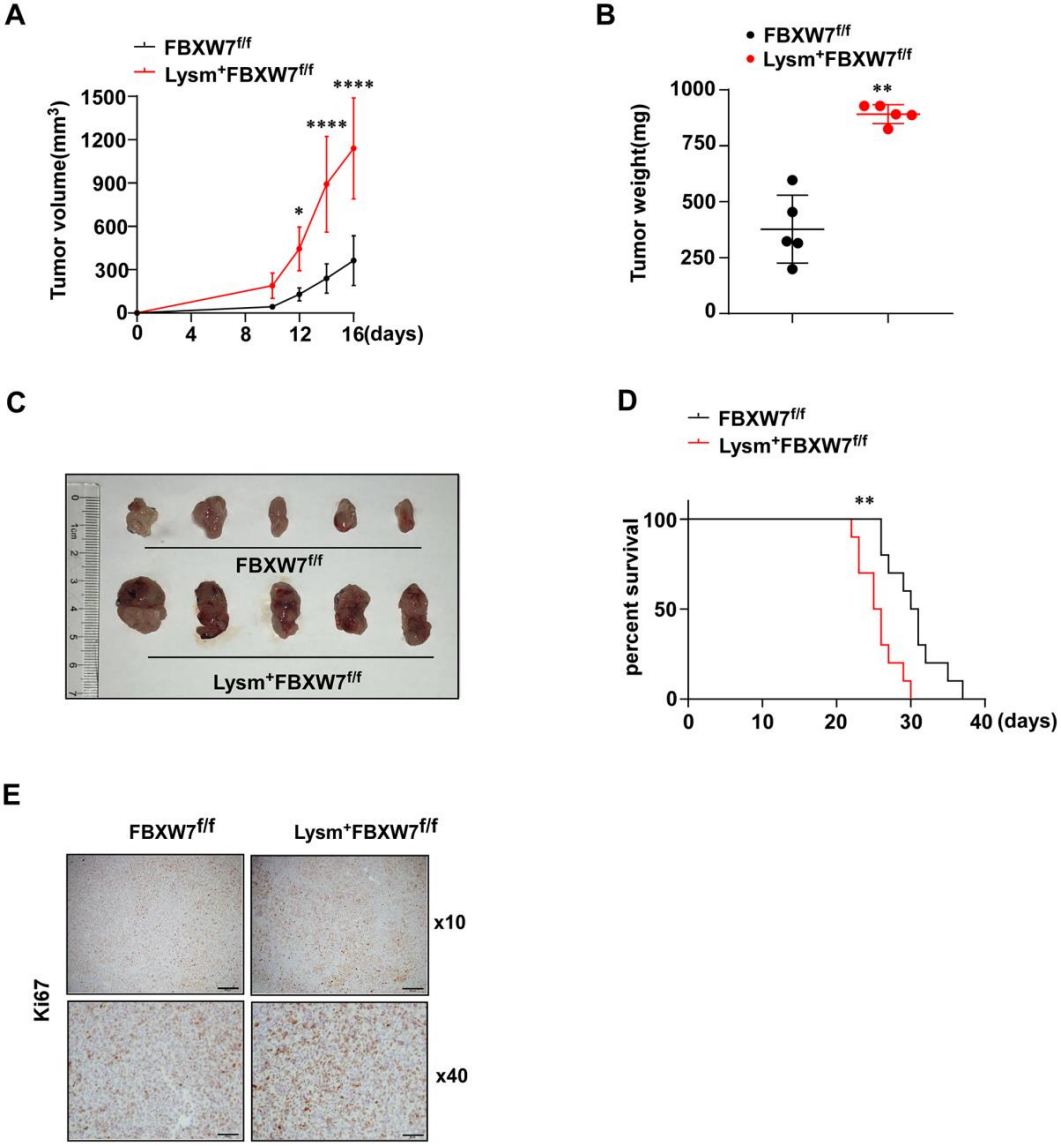
**Lysm^+^FBXW7^f/f^ mice show aggravated tumor progression in an LLC-inoculated model.** (**A**) LLCs were subcutaneously injected into the mice. The volume of tumors from FBXW7^f/f^ and Lysm^+^FBXW7^f/f^ mice were measured and recorded in 16 days (n = 5 per group). (**B**) The weight of tumors at 16 days from both groups (n = 5 per group). (**C**) The appearance of tumors dissected 16 days after inoculation in FBXW7^f/f^ and Lysm^+^FBXW7^f/f^ mice. (**D**) The survival curve of tumor-bearing FBXW7^f/f^ and Lysm^+^FBXW7^f/f^ mice (n = 10 per group). (**E**) Representative images of tumors from two groups immunohistochemically stained with an antibody against Ki67. Scale bars: 10× was 200 μm, 40× was 50 μm. Data are expressed as the mean ± SD and are representative of three independent experiments. *P < 0.05; **P < 0.01; ****P < 0.0001 (two-way ANOVA (**A**), Student’s t test (**B**) and log rank test (**D**)).

Next, we injected LLCs subcutaneously into the flanks of the FBXW7^f/f^ and Lysm^+^FBXW7^f/f^ mice. Tumor volume change was recorded 16 days after the injection of LLCs and was used to evaluate tumor growth. We observed increased tumor volume and accelerated tumor growth in Lysm^+^FBXW7^f/f^ mice (Figure 1A). At 16 days after injection, tumors were dissected, weighed and photographed. We found that the tumor weight was significantly increased in mice with FBXW7 knockout in myeloid cells (Figure 1B). The appearance of the tumors in two groups was evaluated (Figure 1C) and indicated that FBXW7 depletion in myeloid cells promoted tumor development. To analyze the long-term effects of FBXW7 knockout in myeloid cells on tumor prognosis, the survival rates of tumor-bearing mice were recorded. Early death was observed more commonly in Lysm^+^FBXW7^f/f^ mice than in FBXW7^f/f^ mice (Figure 1D). We further analyzed the rates of tumor cell proliferation and found that Ki67 was more highly expressed in Lysm^+^FBXW7^f/f^ mice than in FBXW7^f/f^ mice (Figure 1E). Taken together, these results indicate that the Lysm^+^FBXW7^f/f^ mice show enhanced tumor progression.

### FBXW7 depletion in myeloid cells promotes cancer development by facilitating M2-like TAM polarization

**Figure 2:**
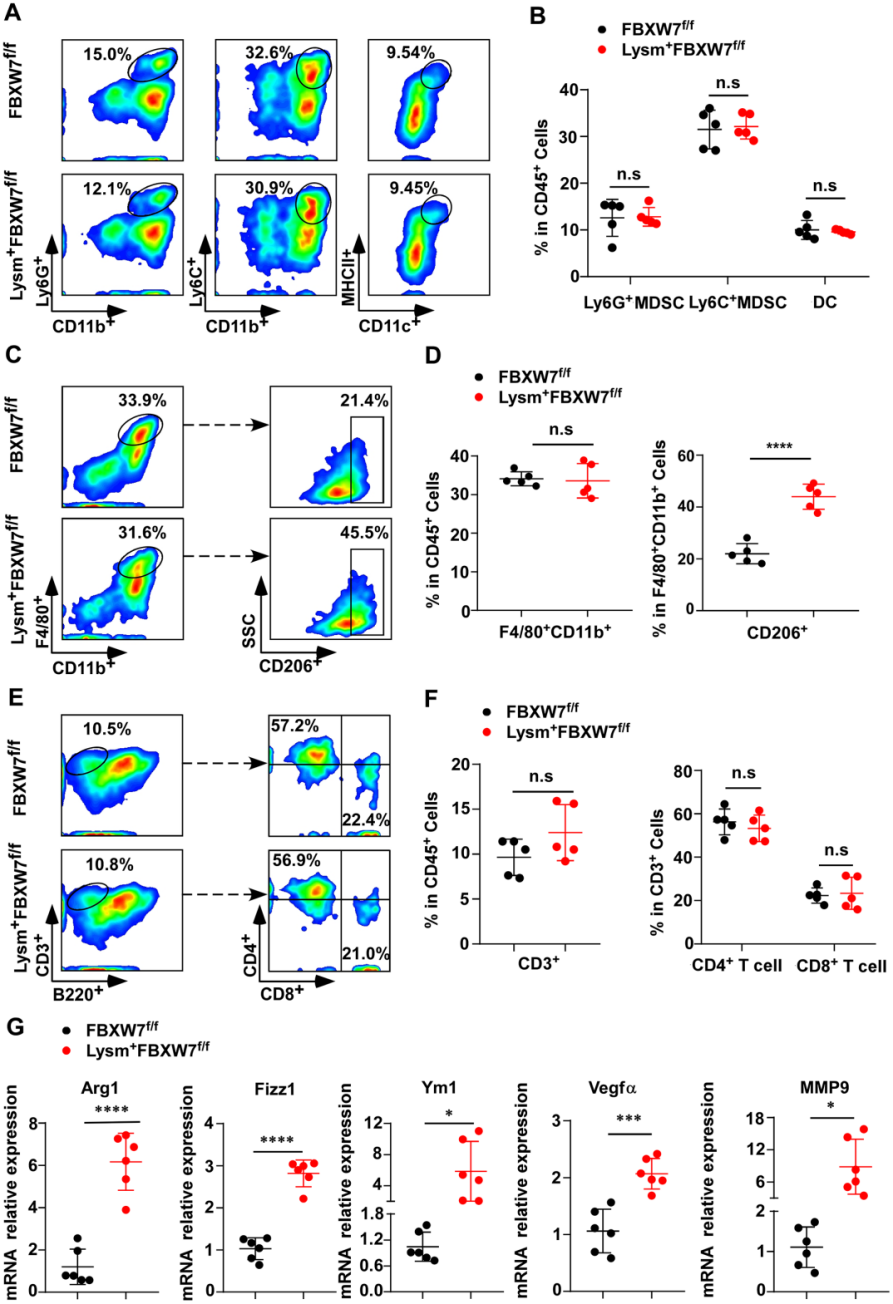
**FBXW7 knockout in myeloid cells remodels the tumor immune microenvironment by promoting M2-like TAM polarization.** (**A**) Tumors were digested to obtain single-cell suspensions. Ly6G^+^CD11b^+^ myeloid-derived suppressor cells (MDSCs), Ly6C^+^CD11b^+^ MDSCs, and MHCII^+^CD11c^+^ dendritic cells in the tumors were analyzed by flow cytometry. (**B**) Statistical analysis of the results in (**A**) (n = 5 per group). (**C**, **D**) Flow cytometry analysis (**C**) and statistical analysis (**D**) of F4/80^+^CD11b^+^ macrophages and CD206^+^ macrophages in tumors (n = 5 per group). (**E**, **F**) Flow cytometry analysis (**E**) and statistical analysis (**F**) of CD3^+^ T cells infiltrating the tumor, CD4^+^ and CD8^+^ T cells in CD3^+^ T cells (n = 5 per group). (**G**) Relative mRNA expression of *Arg1, Fizz1, Ym1, VEGFα,* and *MMP9* in tumors was measured by qRT-PCR (n = 6 per group). Data are shown as the mean ± SD and are representative of three independent experiments. *P < 0.05; ***P < 0.001; ****P < 0.0001; n.s, no significance (Student’s t test (**B**, **D**, **F**, **G**)).

Tumor development is a complicated process that involves many interactions between tumor cells and the tumor microenvironment. At the onset of tumorigenesis, innate and adaptive immune cells are recruited to construct a tumorigenic immune microenvironment [31–34]. To investigate which kind of tumorigenic immune microenvironment compositions promoted tumor progression in Lysm^+^FBXW7^f/f^ mice, we compared the proportion of immune cells infiltrating the tumor tissues of FBXW7^f/f^ and Lysm^+^FBXW7^f/f^ mice by flow cytometry. In innate immune cells, the percentage of Ly6C^+^CD11b^+^ myeloid-derived suppressor cells (MDSCs), Ly6G^+^CD11b^+^ MDSCs, and MHCII^+^CD11c^+^ dendritic cells were comparable between the two groups (Figure 2A, 2B). This suggested that these cells were not the cause of the difference in tumor progression observed between FBXW7^f/f^ and Lysm^+^FBXW7^f/f^ mice.

Macrophages are the most abundant immune cells in the tumor stroma. Hypoxia, lactate, and other factors in tumor microenvironment promote M2-like TAMs polarization [4]. In turn, M2-like TAMs support tumor growth and metastasis through intercellular communication and by producing mediators to shape the tumor microenvironment [16, 35]. According to our data, although the percentage of macrophages (F4/80^+^CD11b^+^) infiltrating the tumor tissues was no different between FBXW7^f/f^ and Lysm^+^FBXW7^f/f^ mice, a higher percentage of CD206^+^ subset in macrophages was observed in the FBXW7-knockout group (Figure 2C, 2D). These results indicate that conditional FBXW7 knockout may switch the TAM phenotype and facilitate polarization to the M2 subset, thus benefiting tumor growth.

Lymphocytes also serve as important and direct anti-tumor effector cells [36, 37]. We detected CD3^+^, CD4^+^, and CD8^+^ lymphocytes from single-cell suspensions of tumor tissues by flow cytometry. There were no significant differences in the proportion of CD3^+^ T cells, CD4^+^ T cells, and CD8^+^ T cells between FBXW7^f/f^ and Lysm^+^FBXW7^f/f^ mice (Figure 2E, 2F), suggesting that FBXW7 knockout did not influence the proportion of the lymphocytes in tumor tissues.

The neutrophil is one of granulocyte expressing a high level of Lyz2 and contributes significantly to cancer development [33, 38]. To examine whether neutrophils are related to the aggravated tumor progression observed in Lysm^+^FBXW7^f/f^ mice, we depleted neutrophils using an anti-Ly6G Ab (Supplementary Figure 2A, 2B). Anti-Ly6G Ab reduced neutrophil recruitment in tumor tissues but did not abolish the difference in tumor progression between FBXW7^f/f^ and Lysm^+^FBXW7^f/f^ mice (Supplementary Figure 2C–2G). Interestingly, Lysm^+^FBXW7^f/f^ mice showed enhanced tumor progression compared to FBXW7^f/f^ mice, with a higher percentage of CD206^+^ macrophages in the tumor tissue, regardless of neutrophil depletion (Supplementary Figure 2H, 2I). These findings indicated that the function of conditional FBXW7 knockout in tumors might be dependent on M2-like TAMs rather than on neutrophils.

To further determine whether the ablation of FBXW7 was responsible for the increase in M2-like TAMs in the tumor microenvironment, we subsequently detected mRNA expression of the immunosuppressive factors Arginase 1 (*Arg1*), resistin like alpha (*Fizz1*), and chitinase-like 3 (*Ym1)* as markers of M2-polarized macrophages in tumor tissues. Higher levels of *Arg1*, *Fizz1*, and *Ym1* were found in the Lysm^+^FBXW7^f/f^ mice compared to the FBXW7^f/f^ mice (Figure 2G). Next, we analyzed changes in the expression of vascular endothelial growth factor α (*VEGFα*) and matrix metalloproteinase 9 (*MMP9*), which are produced by M2-like TAMs to support tumor growth by inducing neovascularization and modifying the extracellular matrix (ECM) [39, 40]. Both factors showed higher levels in the Lysm^+^FBXW7^f/f^ mice compared to the FBXW7^f/f^ mice (Figure 2G). Collectively, these data demonstrate that FBXW7 knockout promotes cancer development by facilitating M2-like TAM polarization to modify the microenvironment.

### FBXW7 knockout facilitates M2 macrophage polarization *in vitro*

**Figure 3:**
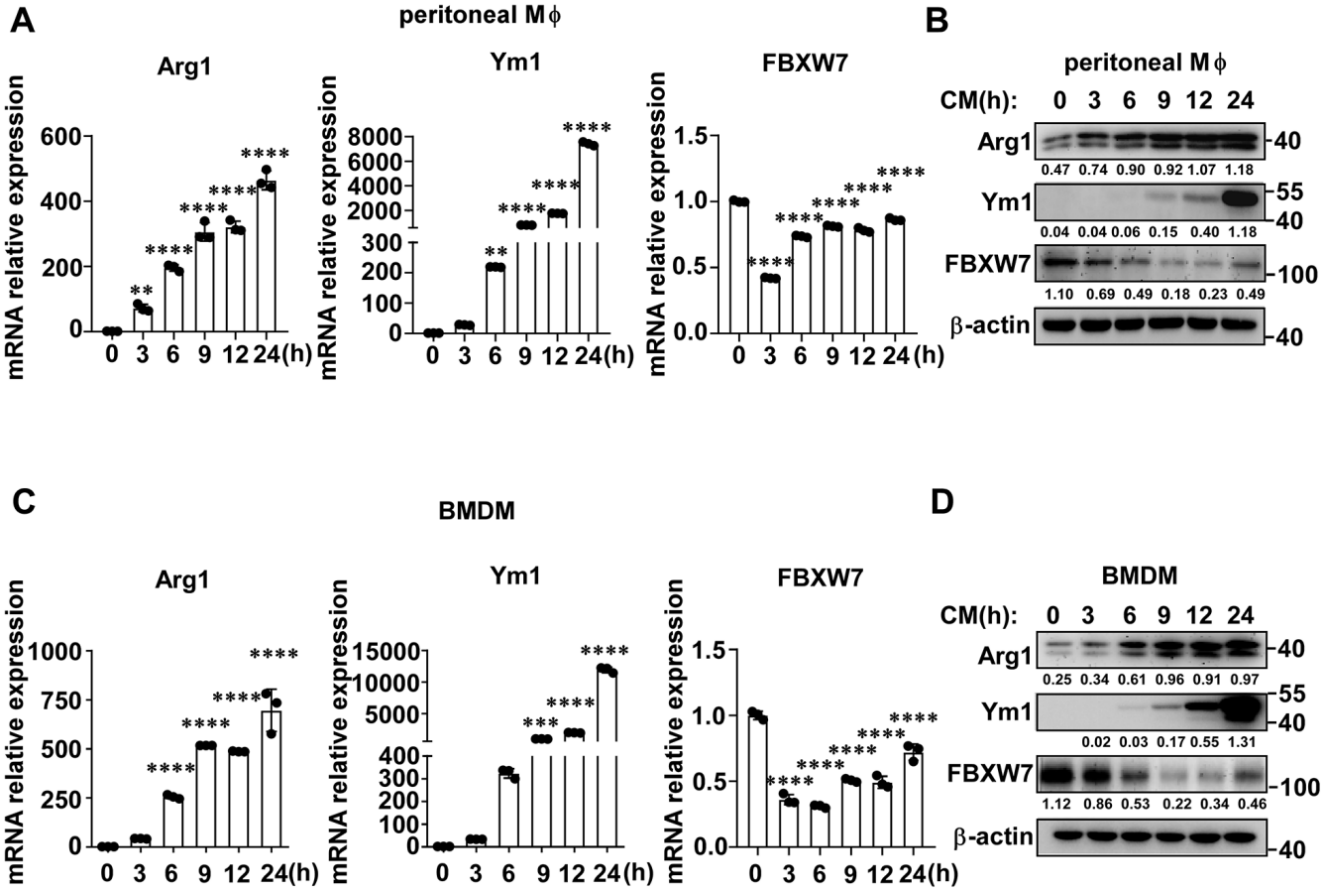
**FBXW7 expression decreases in M2-like TAMs.** (**A**, **B**) Peritoneal macrophages were stimulated with conditioned medium containing LLC supernatant for the indicated time periods. The mRNA (**A**) and protein (**B**) expression levels of Arg1, Ym1, and FBXW7 were measured by qRT-PCR and immunoblotting, respectively. (**C**, **D**) BMDMs were incubated in conditioned medium, and the mRNA (**C**) and protein (**D**) expression levels of Arg1, Ym1, and FBXW7 were measured by qRT-PCR and immunoblotting. Data are shown as the mean ± SD and are representative of three independent experiments. n=3. **P < 0.01; ***P < 0.001; ****P < 0.0001 (one-way ANOVA (**A**, **C**)).

To further determine the role of FBXW7 in the polarization of M2-like TAMs, we stimulated macrophages with LLC supernatant to mimic the lung cancer condition and detected the expression of FBXW7 in M2 macrophages. The gene and protein expression of Arg1 and Ym1, markers of the M2 phenotype, gradually increased in peritoneal macrophages over time. Simultaneously, FBXW7 expression decreased at both the mRNA and protein levels in early time (Figure 3A, 3B). Similar results were observed in bone marrow derived macrophages (BMDMs) (Figure 3C, 3D). Therefore, we assumed that the expression of FBXW7 played a role in the regulation of M2 macrophage polarization.

**Figure 4:**
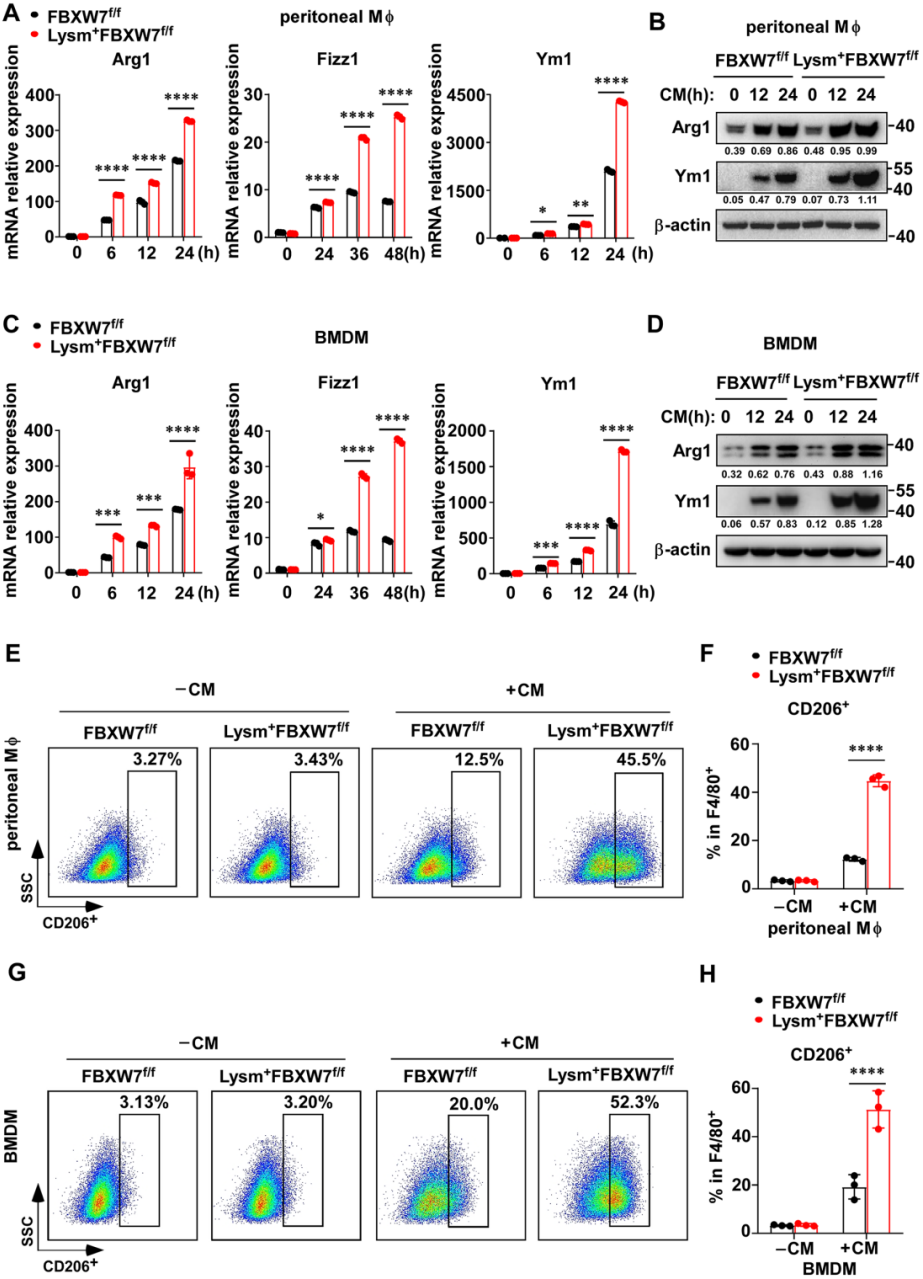
**FBXW7 knockout facilitates M2 macrophage polarization in a tumor microenvironment-mimicking condition.** (**A**) Peritoneal macrophages extracted from FBXW7^f/f^ and Lysm^+^FBXW7^f/f^ mice were treated with conditioned medium containing LLC supernatant. The mRNA expression of *Arg1*, *Fizz1*, and *Ym1* were analyzed by qRT-PCR. (**B**) The protein levels of Arg1 and Ym1 in wild-type and FBXW7-knockout peritoneal macrophages were detected by immunoblotting after conditioned medium stimulation. (**C**, **D**) The mRNA (**C**) and protein (**D**) expression of Arg1, Fizz1, and Ym1 were analyzed by qRT-PCR and western blotting, respectively, in BMDMs incubated with conditioned medium. (**E**, **F**) Flow cytometry analysis (**E**) and statistical analysis (**F**) of the percentage of M2 macrophages (CD206^+^) in wild-type and FBXW7-knockout peritoneal macrophages stimulated with conditioned medium (n = 3 per group). (**G**, **H**) Flow cytometry analysis (**G**) and statistical analysis (**H**) of the percentage of M2 macrophages (CD206^+^) in wild-type and FBXW7-knockout BMDMs stimulated with conditioned medium (n = 3 per group). Data are shown as the mean ± SD and are representative of three independent experiments. *P < 0.05; **P < 0.01; ***P < 0.001; ****P < 0.0001 (two-way ANOVA (**A**, **C**, **F**, **H**)).

To further confirm this hypothesis, we treated peritoneal macrophages and BMDMs from FBXW7^f/f^ and Lysm^+^FBXW7^f/f^ mice with LLC supernatant. We found that higher mRNA levels of *Arg1*, *Fizz1*, and *Ym1* were expressed in FBXW7-knockout peritoneal macrophages compared to the wild-type peritoneal macrophages (Figure 4A). At the protein level, Arg1 and Ym1 levels were increased to a greater extent due to FBXW7 knockout, as confirmed by immunoblotting (Figure 4B). Similar results were observed in BMDMs under the same conditions (Figure 4C, 4D). Subsequently, we performed F4/80 and CD206 staining to examine the proportion of CD206^+^ M2 macrophages in stimulated peritoneal macrophages and BMDMs by flow cytometry. The percentage of CD206^+^ M2 macrophages was higher in FBXW7-knockout macrophages than in wild-type macrophages (Figure 4E–4H). Altogether, our results revealed that FBXW7 knockout facilitated M2 macrophage polarization after incubation with LLC supernatant, consistent with the observed function of FBXW7 in our LLC-inoculated mouse model.

As reported in previous studies, macrophage polarization is triggered by multiple stimuli [3, 41]. In addition to LLC supernatant, we used IL-4, a classical M2 macrophage-inducing factor, to polarize macrophages derived from FBXW7^f/f^ and Lysm^+^FBXW7^f/f^ mice and compared M2 markers expression. The gene and protein expression of Arg1, Fizz1, and Ym1 were substantially higher in FBXW7-knockout M2 macrophages under IL-4 stimulation (Supplementary Figure 3A–3D). Flow cytometry analysis also demonstrated that FBXW7 knockout promoted IL-4-induced M2 macrophage polarization, with a significantly higher proportion of CD206^+^ macrophages (Supplementary Figure 3E–3H). Taken together, FBXW7 knockout enhances M2-associated gene expression at both the mRNA and protein levels following multiple stimuli.

We also analyzed the regulation of FBXW7 in the human monocyte cell line, THP-1. We silenced FBXW7 with small interfering RNA (siRNA) in THP-1 cells and found significantly increased expression of the M2 macrophage markers; Arg1, CD163, transforming growth factor β (TGFβ), and IL10 in FBXW7-silenced THP1 cells after A549 supernatant treatment compared to the unsilenced THP-1 cells (Supplementary Figure 4A–4E).

These data demonstrate that FBXW7 plays an integral role in M2 macrophage polarization.

### FBXW7 knockout promotes expression of pro-tumoral factors in macrophages

**Figure 5:**
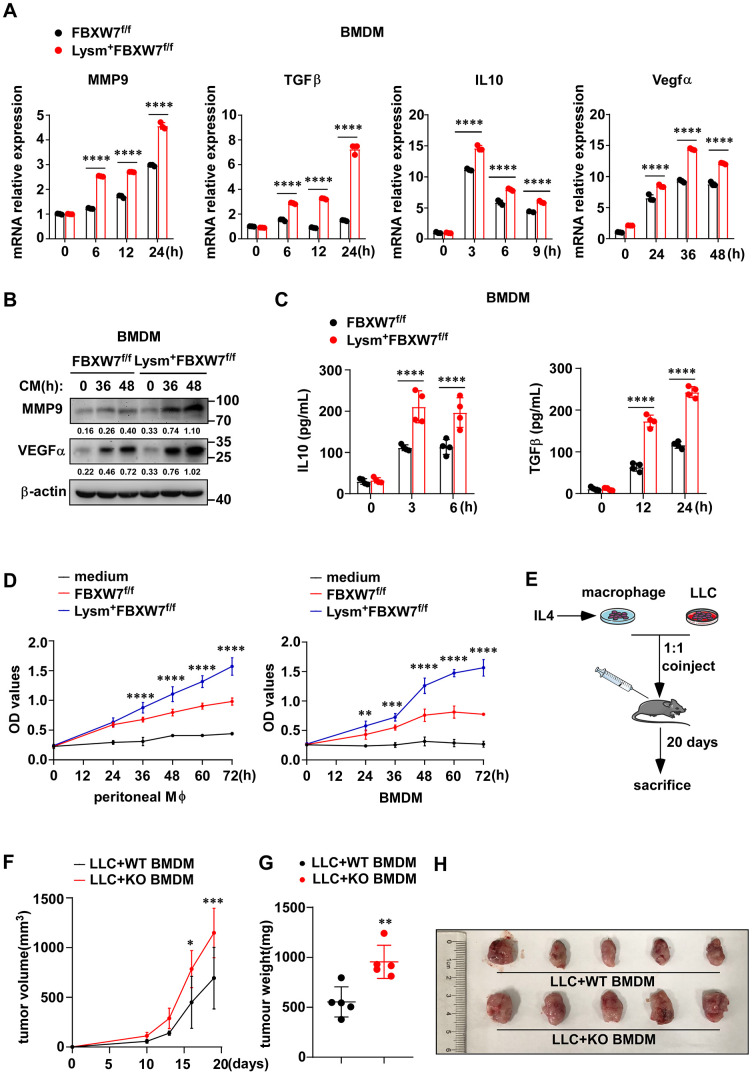
**FBXW7 knockout promotes macrophage expressing pro-tumoral factors.** (**A**) BMDMs from FBXW7^f/f^ and Lysm^+^FBXW7^f/f^ mice were stimulated with conditioned medium, and the mRNA expression of *MMP9*, *IL-10, TGFβ*, and *VEGFα* was examined by qRT-PCR. (**B**) The protein expression of MMP9 and VEGFα in BMDMs incubated with conditioned medium were detected by immunoblotting. (**C**) The protein levels of IL10 and TGFβ in the supernatant of BMDMs which co-cultured with LLCs for indicated time were measured by ELISA kit. (**D**) LLCs were cultured in serum-free RPMI-1640, supernatant from IL-4-induced wild-type or FBXW7-knockout macrophages. The proliferation of LLCs in three groups was measured by MTT assay. (**E**) Schematic representation of the co-injection experimental approach. IL4-induced BMDMs derived from FBXW7^f/f^ and Lysm^+^FBXW7^f/f^ mice were mixed with LLCs at a ratio of 1:1 and injected subcutaneously into healthy wild-type C57BL/6 mice. (**F**, **G**) The volume (**F**) and weight (**G**) of tumors in co-injection mice. (**H**) The appearance of tumors in two groups inoculated with a mixture of M2 macrophages and LLCs. Data are shown as the mean ± SD and are representative of three independent experiments. *P < 0.05; **P < 0.01; ***P < 0.001; ****P < 0.0001 (two-way ANOVA (**A**, **C**, **D**, **F**) and Student’s t test (**G**)).

M2-like TAMs contribute to tumor progression by expressing VEGFα to benefit angiogenic process [42, 43], producing MMP9 to promote tumor invasion [40], secreting IL-10 and TGFβ to maintain an immunosuppressive microenvironment [44]. Therefore, we further investigated whether FBXW7 affected the cancer-promoting function of M2 macrophages. We stimulated peritoneal macrophages and BMDMs from FBXW7^f/f^ and Lysm^+^FBXW7^f/f^ mice with LLC supernatant, and examined the expression of these representative pro-tumoral factors. We found that the mRNA levels of *MMP9*, *TGFβ*, *IL-10,* and *VEGFα* increased in both wild-type and FBXW7-knockout macrophages following stimulation. Furthermore, significantly higher mRNA expression of *MMP9*, *TGFβ*, *IL-10,* and *VEGFα* was observed in FBXW7-knockout macrophages, compared to the wild-type macrophages (Figure 5A, Supplementary Figure 5A). The protein levels of MMP9, TGFβ, IL-10, and VEGFα, detected by immunoblotting or ELISA, raised to a greater extent in FBXW7-knockout macrophages than in wild-type macrophages following stimulation (Figure 5B, 5C and Supplementary Figure 5B, 5C). Next, we cultured wild-type and FBXW7-knockout macrophages with IL-4 to induce M2-phenotype polarization and collected their supernatants. LLCs were separately cultured with serum-free RPMI-1640 medium, the supernatant of wild-type or FBXW7-knockout M2 macrophages. We examined the proliferation of LLCs at the indicated time points via MTT assay, and found that the M2 macrophage supernatant clearly facilitated cancer cells proliferation than the medium group. This effect was enhanced with FBXW7 knockout M2 macrophage supernatant than with the wild-type M2 macrophage supernatant (Figure 5D). Therefore, it suggests that FBXW7-knockout promotes pro-tumoral factors production in M2 macrophages and aggravates tumor progression upon M2 polarization.

To further determine whether the effect of FBXW7 knockout on M2 macrophage polarization was indeed responsible for tumor progression, we subcutaneously injected wild-type C57BL/6 mice with a mixture of LLCs and FBXW7^f/f^ or Lysm^+^FBXW7^f/f^ M2-polarized macrophages at a 1:1 ratio (Figure 5E). We recorded the tumor volume at different time points and the tumor weight at 19 days. Co-injection with LLCs and FBXW7-knockout M2 macrophages resulted in larger tumor volume (Figure 5F) and heavier tumors (Figure 5G), which was also confirmed visually (Figure 5H).

Altogether, these results illustrate that FBXW7 knockout promotes M2 macrophage polarization and leads to the production of cancer-promoting factors by M2 macrophages.

### The inhibition of FBXW7 in M2 macrophage polarization is dependent on c-Myc

**Figure 6:**
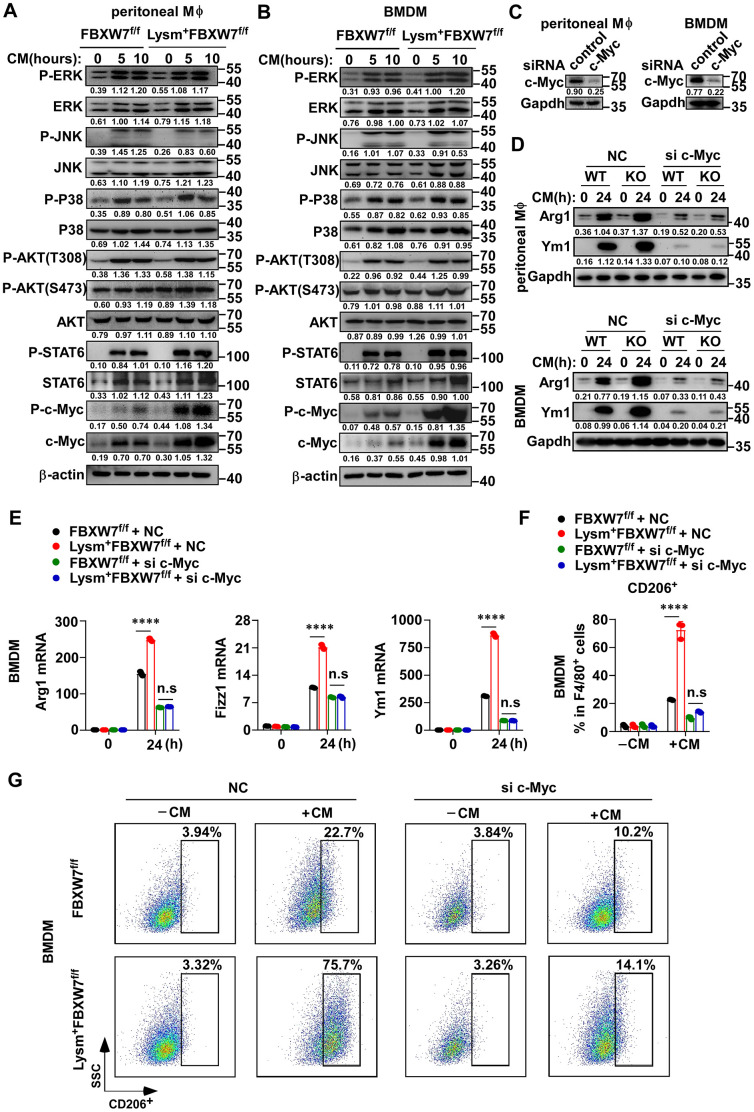
**The role of FBXW7 in regulating M2 macrophage polarization is dependent on c-Myc.** (**A**, **B**) Peritoneal macrophages (**A**) and BMDMs (**B**) derived from FBXW7^f/f^ and Lysm^+^FBXW7^f/f^ mice were stimulated with conditioned medium for the indicated time periods. The phosphorylated or total proteins related to M2 macrophage polarization were analyzed by immunoblotting. (**C**) Immunoblotting was used to analyze the interference efficiency of c-Myc siRNA in primary macrophages following stimulation with conditioned medium for 12 hours. (**D**, **E**) Immunoblotting (**D**) and qRT-PCR analysis (**E**) of Arg1 and Ym1 expression in primary macrophages from FBXW7^f/f^ and Lysm^+^FBXW7^f/f^ mice transfected with or without c-Myc siRNA and stimulated with conditioned medium. (**F**, **G**) Statistical analysis (**F**) and flow cytometry analysis (**G**) of the percentage of M2 macrophages (CD206^+^) in wild-type and FBXW7-knockout macrophages transfected with or without c-Myc siRNA and stimulated with conditioned medium. Data are shown as the mean ± SD and are representative of three independent experiments. ****P < 0.0001; n.s, no significance (two-way ANOVA (**E**, **F**)).

Multiple molecules have been identified as crucial mediators in macrophage polarization, including STAT6, MAPK, AKT, and c-Myc [45–50]. We compared the differences in the expression of these activated downstream signaling molecules in M2 macrophages between wild-type and FBXW7-knockout macrophages after LLC supernatant stimulation. We found that the protein levels of ERK, STAT6, JNK, AKT, and c-Myc were all increased in both groups to some degree after incubation in conditioned medium, and an increase in the phosphorylation of these proteins was also observed. However, their expression and phosphorylation levels were comparable between the two groups, except for c-Myc. We found that c-Myc phosphorylation greatly increased in FBXW7-knockout macrophages stimulated with LLC supernatant (Figure 6A, 6B). Furthermore, the total protein expression of c-Myc was also higher in the FBXW7-knockout group compared to that in the wild-type group (Figure 6A, 6B).

c-Myc is a transcription factor that contributes to cellular growth, cell survival, and cell transformation [51–53], as well as a well-known oncogene. Recently, c-Myc was identified as essential for the polarization of M2-like TAMs in human colon cancer [50]. To further determine the function of c-Myc in M2-like TAMs, and whether c-Myc was responsible for the regulation of FBXW7 in M2 macrophage polarization, we transfected FBXW7^f/f^ or Lysm^+^FBXW7^f/f^ macrophages with siRNA targeting c-Myc, and later stimulated the cells with LLC supernatant. We confirmed the interference efficiency of c-Myc siRNA (Figure 6C) and analyzed the protein and mRNA expression of M2-associated genes. The protein expression of Arg1 and Ym1 significantly decreased due to c-Myc interference in both groups (Figure 6D), consistent with the known positive role of c-Myc in M2 macrophage polarization [50]. Furthermore, c-Myc knockdown using siRNA abolished the discrepancy between the wild-type and FBXW7 knockout macrophages induced by LLC supernatant at the protein levels of Arg1 and Ym1 (Figure 6D). A similar result was observed in terms of the mRNA levels of *Arg1*, *Fizz1*, and *Ym1* (Figure 6E). Next, we compared the proportion of CD206^+^ macrophages in the wild-type and FBXW7-knockout macrophages after c-Myc knockdown and stimulation with conditioned medium. The difference in the proportion of CD206^+^ macrophages caused by FBXW7 knockout was discarded after c-Myc interference (Figure 6F, 6G).

In addition, we detected pro-tumor factors expression of wild-type and FBXW7-knockout macrophages after c-Myc interference. The mRNA and protein expression of MMP9, TGFβ, IL-10, and VEGFα were inhibited with c-Myc knockdown. Likewise, the discrepancy of these factors expression between wild-type and FBXW7-knockout macrophages reduced to some degree (Supplementary Figure 6A–6C). Subsequently, we examined the relationship between c-Myc and FBXW7 in M2 macrophages on LLC proliferation. We collected the supernatant of wild-type and FBXW7-knockout macrophages that transfected with or without c-Myc siRNA to culture LLCs and detected the LLC proliferation rates via MTT assay. The result showed that c-Myc knockdown abolished the difference in the LLC proliferation rates between wild-type and FBXW7-knockout macrophages (Supplementary Figure 6D).

Collectively, these data indicated that the effect of FBXW7 knockout on M2-like TAM polarization and on the cancer-promoting function of M2 macrophages was dependent on c-Myc.

### FBXW7 mediates the ubiquitination and degradation of c-Myc in M2 macrophages

**Figure 7:**
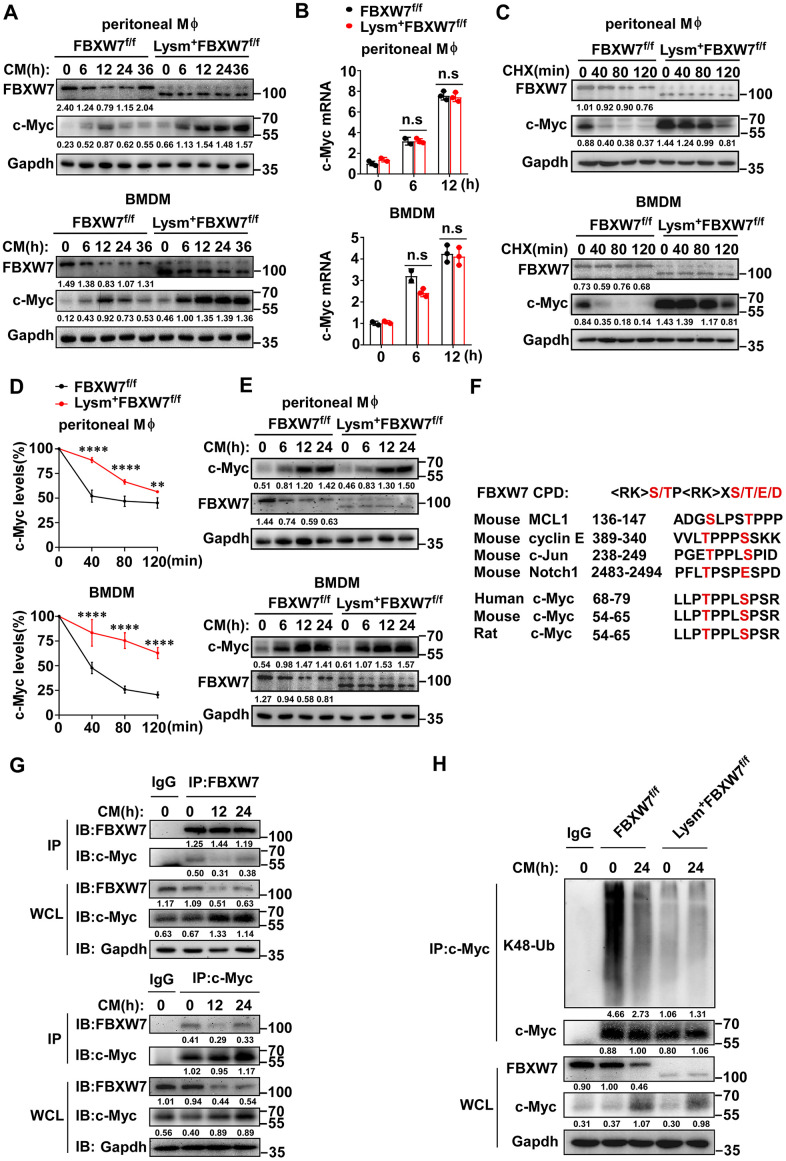
**FBXW7 mediates degradation and ubiquitination of c-Myc in M2-like TAMs.** (**A**) The protein expression of c-Myc and FBXW7 in wild-type and FBXW7-knockout primary macrophages stimulated with conditioned medium were measured by immunoblotting. (**B**) qRT-PCR analysis of *c-Myc* mRNA expression in wild-type and FBXW7-knockout primary macrophages after conditioned medium treatment. (**C**) Immunoblot analysis of c-Myc and FBXW7 in wild-type and FBXW7-knockout primary macrophages treated with CHX (20 μg/mL) for the indicated time period after conditioned medium pre-treatment. (**D**) The quantification of relative c-Myc levels in (**C**). (**E**) The protein expression of c-Myc and FBXW7 in wild-type and FBXW7-knockout primary macrophages after treatment with MG132 (10 μM) and conditioned medium. (**F**) The phosphodegron sequence alignment of c-Myc recognized by FBXW7 with MCL1, cyclin E, Notch1, and c-Jun. The FBXW7 phosphodegron sequence present in c-Myc is conserved across different species. Conserved residues within the degron sequences are shown in red. (**G**) Immunoblot analysis of BMDMs stimulated with conditioned medium for the indicated time periods and treated with MG132, followed by immunoprecipitation with specific antibody-conjugated agarose or immunoglobulin G (IgG)-conjugated agarose. (**H**) Immunoblot analysis of the K48 ubiquitination of c-Myc in wild-type and FBXW7-knockout BMDMs stimulated with conditioned medium. Data are shown as the mean ± SD and are representative of three independent experiments. *P < 0.05; **P < 0.01; ***P < 0.001; ****P < 0.0001; n.s, no significance (two-way ANOVA (**B**, **D**)).

Previous studies have shown that the E3 ubiquitin ligase FBXW7 mediates the ubiquitination and proteasome-dependent degradation of c-Myc [54–56]. Therefore, we investigated whether FBXW7 could influence the stability of c-Myc to regulate M2 macrophage polarization. We analyzed c-Myc expression in M2 macrophages incubated with the LLC supernatant, and found that the levels of c-Myc in the wild-type group increased at first and gradually declined later. However, in FBXW7-knockout M2 macrophages, c-Myc increased and did not decrease over time, and the expression of c-Myc in the FBXW7-knockout group was higher than in the wild-type group at all time points (Figure 7A). In contrast to the c-Myc expression trend, the FBXW7 expression in wild-type macrophages stimulated with LLC supernatant decreased initially and gradually increased at late (Figure 3C, 3D and Figure 7A). This suggested that FBXW7 played a role in c-Myc accumulation in M2 macrophages. We further analyzed the mRNA levels of *c-Myc* in wild-type and FBXW7-knockout M2 macrophages. The mRNA expression of *c-Myc* similarly increased in both groups (Figure 7B), suggesting that FBXW7 depletion exerted an effect on c-Myc at the post-translational level, rather than during transcription.

Next, we treated macrophages with cycloheximide (CHX) to block protein synthesis to analyze the half-life of c-Myc. The half-life of c-Myc was markedly extended in FBXW7-knockout macrophages compared to that in wild-type macrophages (Figure 7C, 7D). Subsequently, we used MG132 to inhibit proteasome-dependent degradation, and found that the difference in protein expression of c-Myc caused by FBXW7 knockout was rescued (Figure 7E). These results indicated that FBXW7 knockout attenuated c-Myc degradation and promoted c-Myc accumulation in M2 macrophages.

The E3 ligase FBXW7 catalyzes the ubiquitination of targeted substrates by recognizing a typical sequence motif called the conserved Cdc4 phosphodegron (CPD) motif. The CPD motif contains phosphorylated threonine or serine residues at positions “0” and “+4” [25]. To further investigate the interaction between FBXW7 and c-Myc, we analyzed the amino acid sequence of c-Myc among different species and found that c-Myc contained the CPD of FBXW7 (Figure 7F). We analyzed the endogenous interactions between FBXW7 and c-Myc by co-immunoprecipitation and found that FBXW7 interacted with c-Myc in M2 macrophages. A similar result was observed in reciprocal co-immunoprecipitation experiments (Figure 7G).

FBXW7 catalyzes the K48-linked polyubiquitination of c-Myc and mediates its degradation via the ubiquitin-proteasome system [54, 55]. Therefore, we examined whether FBXW7 would catalyze c-Myc K48-linked polyubiquitination in M2 macrophages. The FBXW7 knockout resulted in an inhibition of c-Myc K48-linked ubiquitination in M2 macrophages compared to in wild-type (Figure 7H).

In conclusion, these data demonstrated that FBXW7 interacted with c-Myc and catalyzed c-Myc K48-linked polyubiquitination to decrease its stability in M2 macrophages.

## DISCUSSION

In this study, we demonstrate that myeloid cell-specific FBXW7 deficiency can promote tumor progression and increase the proportion of M2-like TAMs in tumor tissues after subcutaneous inoculation with LLCs. We find that FBXW7 plays a critical role in M2 macrophage polarization and pro-tumoral factors production. The inhibition in M2-like TAM polarization caused by FBXW7 occurs through mediating c-Myc degradation via the ubiquitin-proteasome system, by the attachment of K48 poly-ubiquitin chains to c-Myc.

The E3 ligase FBXW7 is a known tumor suppressor. FBXW7 deletion in tumor cells determines the fate of tumor cells through limiting oncoprotein ubiquitin-mediated proteolysis and promoting their aberrant accumulation. FBXW7 also plays a pivotal role in tumorigenesis suppression through other manners. FBXW7 controls stem cells quiescence, self-renewal, and differentiation to influence tumorigenesis [57–60]. FBXW7 knockout in mouse embryonic fibroblasts induces the epithelial-to-mesenchymal transition to promote tumor metastasis [26]. FBXW7 mutations in BMSCs can increase the production of CCL2 to recruit immunosuppressive cells and promote metastasis [27]. We have now demonstrated a new function of FBXW7 in tumorigenesis suppression. FBXW7 depletion in myeloid cells could promote tumor growth. After subcutaneous injection with LLCs, Lysm^+^FBXW7^f/f^ mice had a higher death rate and larger tumor volumes compared to their wild-type littermates. Moreover, these differences were related to an increase in the proportion of CD206^+^ TAMs caused by FBXW7 knockout in tumor tissues.

Cytokines, chemokines, and other mediators produced by M2-like TAMs regulate the immune suppressive microenvironment and modify the tumor microenvironment to facilitate cancer initiation, metastasis, and development [40, 42–44, 61, 62]. As one of the critical determinants in various cellular processes, the role of ubiquitination in macrophage polarization should be recognized. The E3 ligase Trim24 can catalyze acetyltransferase CREB-binding protein (CBP) ubiquitination to facilitate CBP-mediated acetylation of STAT6 to inhibit M2 macrophage polarization [63]. In the present study, we demonstrate that the E3 ligase FBXW7 is a novel mediator of M2 macrophage polarization. We observed that the expression of M2-associated proteins increased in primary macrophages derived from Lysm^+^FBXW7^f/f^ mice upon stimulation with LLC supernatant or IL-4. Furthermore, FBXW7 knockout promoted increased expression of cancer-promoting factors by M2-like TAMs. The percentage of M2-like TAMs infiltrated in tumor tissues in Lysm^+^FBXW7^f/f^ tumor-bearing mice was increased, accompanied with exacerbated tumor growth and a poor prognosis. In addition, Mice co-injected with LLCs and FBXW7-knockout M2 macrophages displayed more severe tumor progression.

c-Myc is a transcription factor that regulates several key signaling pathways involved in cellular growth and proliferation [51]. c-Myc can promote immunosuppression as well as enhance adaptive immune response [64–68]. In the innate immune response, c-Myc plays a role in M2 macrophage polarization. The increased expression of c-Myc has previously been observed in M2 macrophages, and c-Myc can directly interact with the promoters of M2 macrophage-associated genes [50]. Since c-Myc is a downstream effector of multiple signaling pathways, its expression and activity are also controlled by other upstream signaling pathways, which subsequently controls activation and subtype differentiation of the macrophages [69, 70]. In previous studies, FBXW7 has been proven to catalyze the K48-linked ubiquitination of c-Myc, resulting in its degradation, thus regulating the stability of c-Myc to influence cell proliferation and tumorigenesis [54–56].

According to our data, c-Myc expression increased in LLC supernatant-induced macrophages, and this increase in c-Myc expression was affected to some extent by FBXW7. As the incubation time of the LLC supernatant increased, reduced FBXW7 expression attenuated its interaction and ubiquitination of c-Myc, increasing the stability and accumulation of c-Myc, and promoting M2-related gene expression, leading to M2-like TAM polarization. However, in late stages, FBXW7 gradually increased to restore the regulation of c-Myc and prevent excessive or abnormal M2 macrophage polarization. When FBXW7 expression was abrogated, its function in limiting M2 macrophage polarization was abolished, increasing the number of aberrant M2-like TAMs that can promote cancer development.

While our work identified a novel role of FBXW7 in suppressing tumor growth, there are still notable questions that remain to be addressed. In a previous study, FBXW7 mutations in BMSCs, but not in macrophages, were found to be major contributors to the increased production of CCL2 leading to metastasis [27]. Interestingly, while the size of metastasized tumor nodules was reduced by treatment with a CCR2 antagonist, the number of tumor nodules was not affected [27]. This suggests that FBXW7 deficiency in bone marrow cells, including macrophages, MDSCs, and dendritic cells, may be involved in different stages of cancer development and might regulate tumor growth and metastasis through different mechanisms. Our previous study shows that FBXW7 mutations in macrophages may alleviate lung metastasis of murine melanoma, but the detailed mechanisms underlying these results remain unknown [71]. This also suggests that FBXW7 may affect tumor growth and metastasis through different mechanisms via macrophages. Considered that the same molecule or its isoforms may play a different regulatory role in macrophage activation states, several isoforms of FBXW7 may play different regulatory roles during macrophage differentiation and tumor progression. [25, 69, 72–74]. In addition, while the contribution of M2 macrophages has been well identified in tumor progression, the role of M1 macrophages in tumor remains controversial [75–78]. Clarifying the roles of different macrophage subset during cancer progression and at different stages is needed in future works. Additional experiments are required to elucidate the above problems.

In summary, our work demonstrates that FBXW7 mediates c-Myc K48-linked ubiquitination to limit the accumulation of c-Myc, leading to the inhibition of M2-like TAM polarization, thereby exerting a tumor-suppressive effect. These findings improve our understanding of the function of FBXW7 in macrophage plasticity and how it influences cancer development through non-malignant cells.

## MATERIALS AND METHODS

### Antibodies and reagents

For a list of antibodies and reagents used, see Supplementary Table 1.

### Mice

FBXW7^f/f^ mice on a C57BL/6J background were purchased from Jackson Laboratories. Lysm-Cre mice C57BL/6J were kindly provided by Dr. Ximei Wu, Zhejiang University School of Medicine. Lysm^+^FBXW7^f/f^ C57BL/6J mice were generated as we previously described [29]. C57BL/6J mice (6-8 weeks old) were purchased from the Model Animal Research Center of Nanjing University (Nanjing, China). All mice were bred with standard diet and kept at the University Laboratory Animal Center in a specific-pathogen-free environment and used at 6-10 weeks of age. All animal experiments were approved by the Animal Ethics Committee of Zhejiang University in compliance with institutional guidelines.

### Cell culture

LLCs were purchased from the Cell Bank of the Chinese Academy of Science, Shanghai, China, and cultured in Dulbecco’s modified Eagle’s medium (DMEM) with 10% fetal bovine serum (FBS). Mouse peritoneal macrophages were induced by thioglycolate (Merck, Darmstadt, Germany) and extracted by peritoneal lavage. The cells were cultured in RPMI-1640 with 10% FBS. Bone marrow-derived macrophages (BMDMs) were extracted from mice femurs and cultured in RPMI-1640 with 10% FBS supplemented with recombinant mouse macrophage colony-stimulating factor (20 ng/mL).

For cell stimulation, peritoneal macrophages and BMDMs were treated with IL-4 (20 ng/ml) or conditioned medium containing LLC supernatant for the indicated time periods. Conditioned media were prepared as described previously [4].

### Tumor model

Tumors were induced by injecting 1 × 10^6^ LLCs in 100 μl PBS subcutaneously into the right flanks of 8-10-week-old mice. The mice were anesthetized and sacrificed on day 16 or when the largest diameter of the tumor exceeded 20 mm. Tumors were measured with calipers and removed for further experiments. To analyze the survival of tumor-induced mice, 3 × 10^5^ LLCs in 100 μl PBS were injected subcutaneously into the right flanks of 8–10-week-old mice. For the co-injection model, as described previously [79], IL-4-induced M2 macrophages derived from FBXW7^f/f^ or Lysm^+^FBXW7^f/f^ mice were mixed with LLCs at a ratio of 1:1. Then, 2 × 10^6^ mixed cells in 200 μl PBS were co-injected subcutaneously into 6–8-week-old healthy wild-type C57BL/6 mice. The tumor volume (mm3) was calculated as following formula: volume = 0.5 x length x width^2^.

### Neutrophil depletion

As described previously [80], we injected 5.5 μg/g rat IgG2a isotype control antibody or 5.5 μg/g anti-Ly6G antibody intraperitoneally for 24 h prior to inoculating LLC cells. This was repeated every 3 days throughout the study. Collected blood samples from mice tail vein and analyzed the proportion of neutrophils to evaluate the efficiency of neutrophil depletion.

### Flow cytometry analysis

Single-cell suspensions from tumors were prepared as described previously [81]. Briefly, the tumors were minced with scissors and digested in serum-free RPMI-1640 supplemented with Liberase (50 μg/ml) and DNase I (1 μg/ml) at 37° C for 30 min. The single-cell suspensions were obtained through filtering, removing red blood cells, and washing with PBS after digestion. The prepared single-cell suspensions were then stained with the indicated antibodies and analyzed by flow cytometry.

### Immunohistochemistry

Tumor tissues were fixed in 10% phosphate-buffered formalin, embedded in paraffin, sectioned, and stained with the corresponding antibodies according to the manufacturers’ protocols.

### Quantitative reverse transcriptase PCR

Total RNA was isolated from cells or tissues using Trizol reagent (Takara Bio, Shiga, Japan) and reverse transcribed to cDNA using ReverTra Ace (Toyobo, Osaka, Japan). For quantitative PCR (qPCR), cDNA fragments were amplified with the SYBR Green master Rox kit (Takara Bio). All protocols were performed in accordance with the manufacturers’ protocols. The primer sequences used are shown in Supplementary Table 2 and Supplementary Table 3.

### ELISA assay

Macrophages and tumor cells were co-cultured in Transwell chambers with 8-μm membrane pores in a 24-well plate in serum-free RPMI-1640 medium for indicated times. Collected macrophage supernatant and detected the cytokines expression using ELISA kits according to the manufacturer’s instructions.

### Co-immunoprecipitation and immunoblot analysis

For co-immunoprecipitation, cells were collected and lysed with IP Lysis Buffer (Pierce Biotechnology, Waltham, MA, USA) supplemented with protease inhibitor for 30 min at 4° C. Cell lysates were centrifuged for 10 min at 12,000 × *g*, and the supernatants of the centrifuged cell lysates were incubated with specific antibodies bound to protein G magnetic beads overnight at 4° C. Then, the beads were washed three times with IP washing buffer and eluted with SDS loading buffer. Finally, equal amounts of eluted immunoprecipitants were subjected to immunoblot analysis.

### Western blotting

The cells were lysed with cell lysis buffer (Cell Signaling Technology) supplemented with protease inhibitor. The concentration of supernatants from the centrifuged cell lysates were measured using the bicinchoninic acid (BCA) assay. Equal amounts of each protein sample were subjected to SDS-PAGE and electrically transferred to a polyvinylidene fluoride membrane (Millipore, Billerica, MA, USA). Immunoblots were incubated with the corresponding antibodies, as described above. Protein bands were visualized using the Pierce enhanced chemiluminescence (ECL) kit and then visualized and quantified using the Image J software.

### MTT assay

Briefly, LLCs (3 × 10^3^/well) were plated in 96-well plates and incubated in serum-free RPMI-1640 for 24 h. Then, the cells were stimulated with supernatant collected from M2 macrophages for the indicated time periods, prior to the addition of 10 μl MTT reagent to each well and incubation for 4 h. Next, 100 μl DMSO was added to each well and the plates were incubated with shaking for 10 min. The optical density value was detected using a Model 680 microplate reader (Bio-Rad Laboratories, Hercules, CA, USA).

### Cell transfection

siRNAs were designed for *c-Myc* and *FBXW7* gene knockdown. Both siRNAs were produced by GenePharma (Shanghai, China). The sequences used are shown in Supplementary Table 1. Cells were transfected with the indicated siRNAs using Lipofectamine RNAiMAX Reagent (Invitrogen, Carlsbad, CA, USA) according the manufacturer’s protocol. The knockdown efficiency of siRNAs was tested by western blot analysis.

### Statistical analysis

All data were analyzed by GraphPad Prism 8 software. All data are shown as the mean ± SEM or mean ± SD. A two-tailed, unpaired Student’s *t*-test was used for comparisons between two groups, and two-way ANOVA was used for multiple comparisons. The log-rank (Mantel-Cox) test was used to determine statistical significance in the mouse survival study. A value of *P* < 0.05 was considered statistically significant.

## Supplementary Material

Supplementary Figures

Supplementary Tables
