# Supplementary material for: E3 ligase FBXW7 restricts M2-like tumor-associated macrophage polarization by targeting c-Myc
Source: Aging (Albany NY). 2020 Dec 1;12(23):24394–423. doi: 10.18632/aging.202293 (PMC7762499; doi:10.18632/aging.202293)
Supplement: Supplementary Figures [file aging-12-202293-s001.pdf]

SUPPLEMENTARY FIGURES

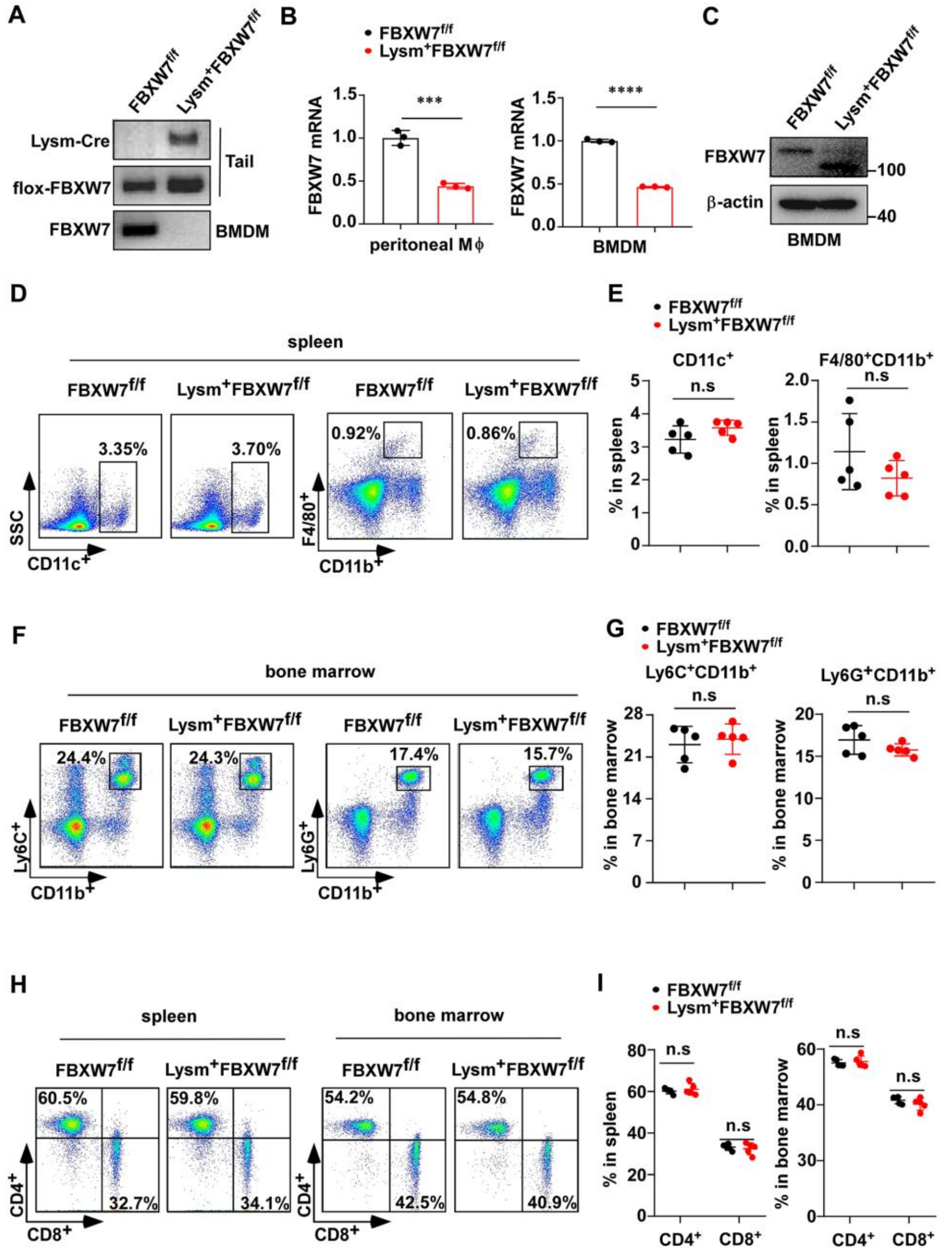

Supplementary Figure 1. FBXW7 knockout in myeloid cells does not affect the development of myeloid cells and lymphocytes. (**A**) PCR analysis “Lysm-Cre”, “flox” sequences of FBXW7$^{f/f}$ and Lysm^{+}FBXW7$^{f/f}$ mice, PCR analysis the excision of exons 5 and 6 of FBXW7 in bone marrow-derived macrophages (BMDMs) from FBXW7$^{f/f}$ and Lysm^{+}FBXW7$^{f/f}$ mice. (**B**) qRT-PCR analysis of *FBXW7* expression in peritoneal macrophages and BMDMs derived from FBXW7$^{f/f}$ and Lysm^{+}FBXW7$^{f/f}$ mice. (**C**) Western blot analysis of FBXW7 expression in BMDMs derived from FBXW7$^{f/f}$ and Lysm^{+}FBXW7$^{f/f}$ mice. (**D**, **E**) Flow cytometry analysis (**D**) and statistical analysis (**E**) of CD11c^{+} dendritic cells and F4/80^{+}CD11b^{+} macrophages in the spleen of FBXW7$^{f/f}$ and Lysm^{+}FBXW7$^{f/f}$ mice (n=5 per group). (**F**, **G**) Flow cytometry analysis (**F**) and statistical analysis (**G**) of Ly6C^{+}CD11b^{+} monocytes and Ly6G^{+}CD11b^{+} granulocytes in the bone marrow of FBXW7$^{f/f}$ and Lysm^{+}FBXW7$^{f/f}$ mice (n=5 per group). (**H**) Flow cytometry analysis of CD4^{+} and CD8^{+} T cells in the spleen and bone marrow from FBXW7$^{f/f}$ and Lysm^{+}FBXW7$^{f/f}$ mice. (**I**) Statistical differences in the proportions of CD4^{+} and CD8^{+} T cells in the spleen and bone marrow of FBXW7$^{f/f}$ and Lysm^{+}FBXW7$^{f/f}$ mice by flow cytometry (n=5 per group). Data are shown as the mean ± SD and are representative of three independent experiments. n = 3 or 5. ***$P < 0.001$; ****$P < 0.0001$; n.s, no significance (Student’s t test (**B**, **E**, **G**, **I**)).

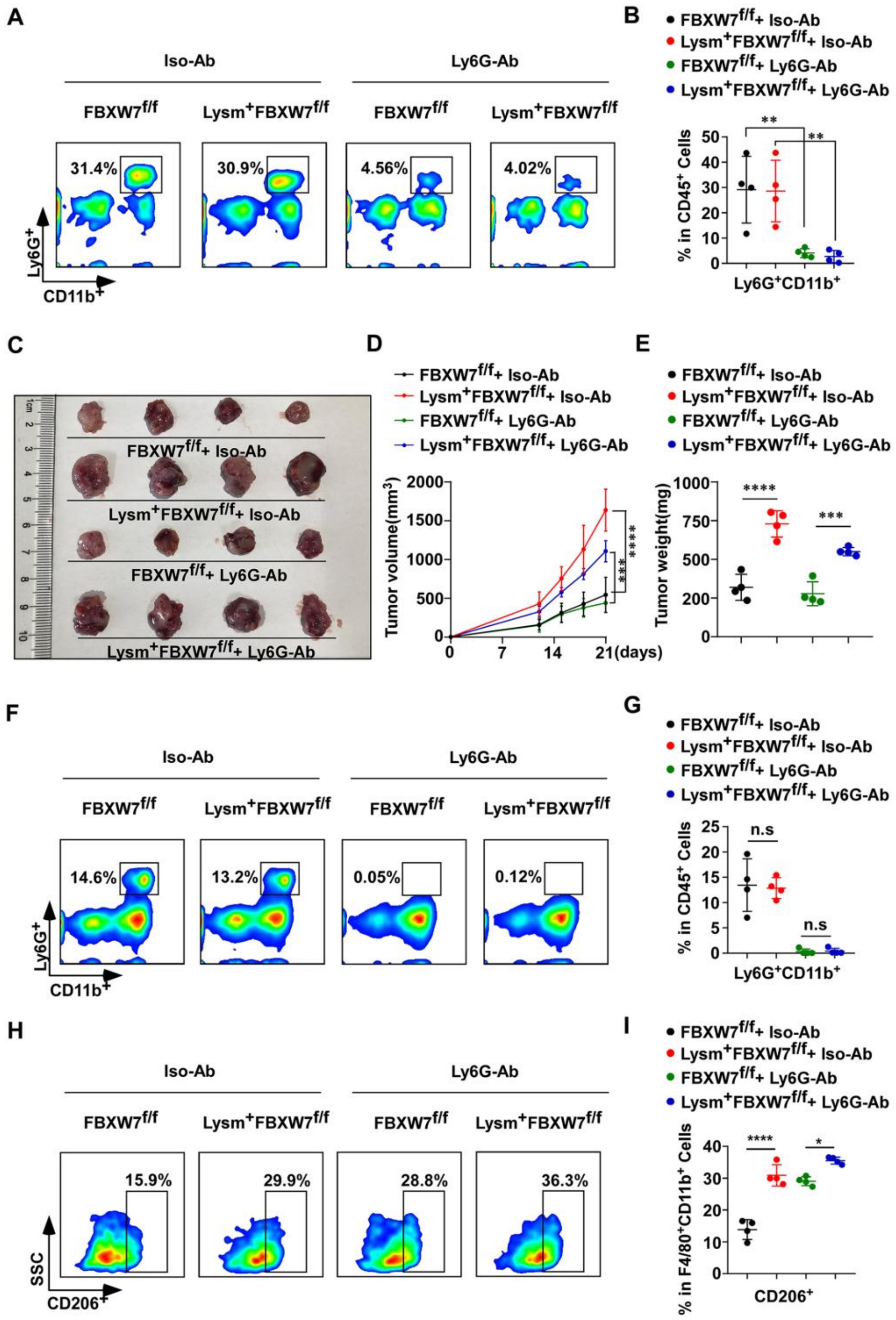

Supplementary Figure 2. Neutrophils are not responsible for the aggravated tumor progression in Lysm$^+$FBXW7$^{f/f}$ mice. (**A**, **B**) Neutrophils were depleted by anti-Ly6G mAb. Flow cytometry analysis (**A**) and statistical analysis (**B**) of the percentage of neutrophils (Ly6G$^+$CD11b$^+$) in the blood of FBXW7$^{f/f}$ and Lysm$^+$FBXW7$^{f/f}$ mice with or without anti-Ly6G mAb used showed to evaluate the efficiency of depletion. (n = 4 per group). (**C**–**E**) FBXW7$^{f/f}$ and Lysm$^+$FBXW7$^{f/f}$ mice inoculated with LLCs after anti-Ly6G mAb or isotype control antibody used. The appearance (**C**), volume (**D**), weight (**E**) of tumors in four groups in 21 days. (**F**, **G**) Flow cytometry analysis (**F**) and statistical analysis (**G**) of the percentage of neutrophils (Ly6G$^+$CD11b$^+$) in the tumors of FBXW7$^{f/f}$ and Lysm$^+$FBXW7$^{f/f}$ mice with or without anti-Ly6G mAb used (n = 4 per group). (**H**, **I**) Flow cytometry analysis (**H**) and statistical analysis (**I**) of the percentage of CD206$^+$ macrophages in tumors of FBXW7$^{f/f}$ and Lysm$^+$FBXW7$^{f/f}$ mice with or without anti-Ly6G mAb used (n = 4 per group). *P < 0.05; **P < 0.01; ***P < 0.001; ****P < 0.0001; n.s, no significance (one-way ANOVA (**B**, **D**, **E**, **G**, **I**)).

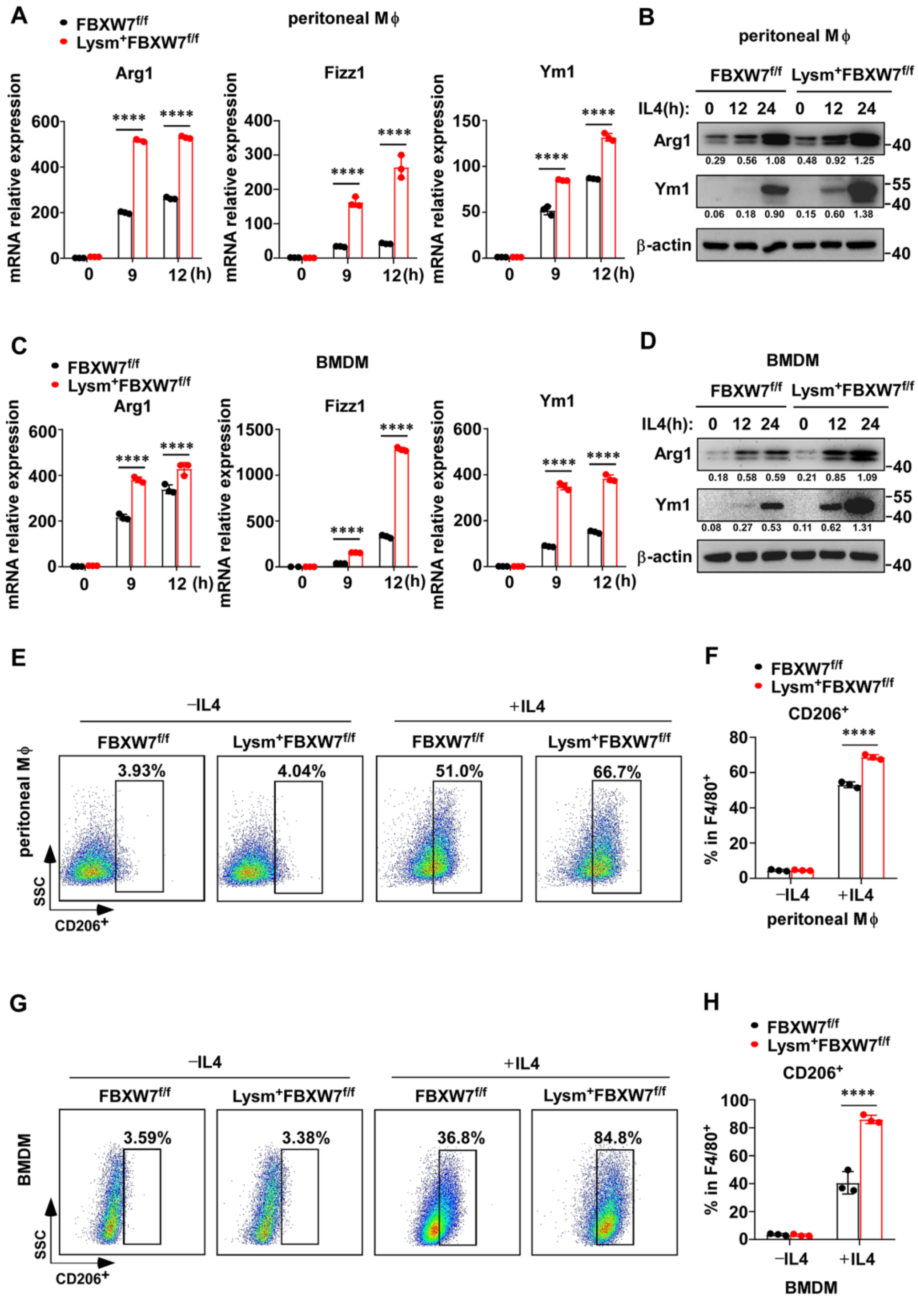

Supplementary Figure 3. FBXW7 knockout enhances IL-4-induced M2 macrophage polarization. (**A**) Peritoneal macrophages extracted from FBXW7$^{f/f}$ and Lysm^{+}FBXW7$^{f/f}$ mice were treated with IL-4 (20 ng/ml), and the mRNA expression of *Arg1*, *Fizz1*, and *Ym1* was analyzed by qRT-PCR. (**B**) The protein expression of Arg1 and Ym1 from wild-type and FBXW7-knockout peritoneal macrophages stimulated with IL-4 was detected by immunoblotting. (**C**, **D**) The mRNA (**C**) and protein (**D**) expression of M2-like TAM-associated genes were analyzed by qRT-PCR and immunoblotting, respectively, in BMDMs stimulated with IL-4. (**E**, **F**) Flow cytometry analysis (**E**) and statistical analysis (**F**) of the percentage of M2 macrophages (CD206^{+}) in wild-type and FBXW7-knockdown peritoneal macrophages after IL-4 stimulation for 24 h (n = 3 per group). (**G**, **H**) Flow cytometry analysis (**G**) and statistical analysis (**H**) of the percentage of M2 macrophages (CD206^{+}) in wild-type and FBXW7-knockout BMDMs after IL-4 stimulation for 24 h (n = 3 per group). Data are shown as the mean ± SD and are representative of three independent experiments. ***$P < 0.001$; ****$P < 0.0001$; n.s, no significance (two-way ANOVA (**A**, **C**, **F**, **H**)).

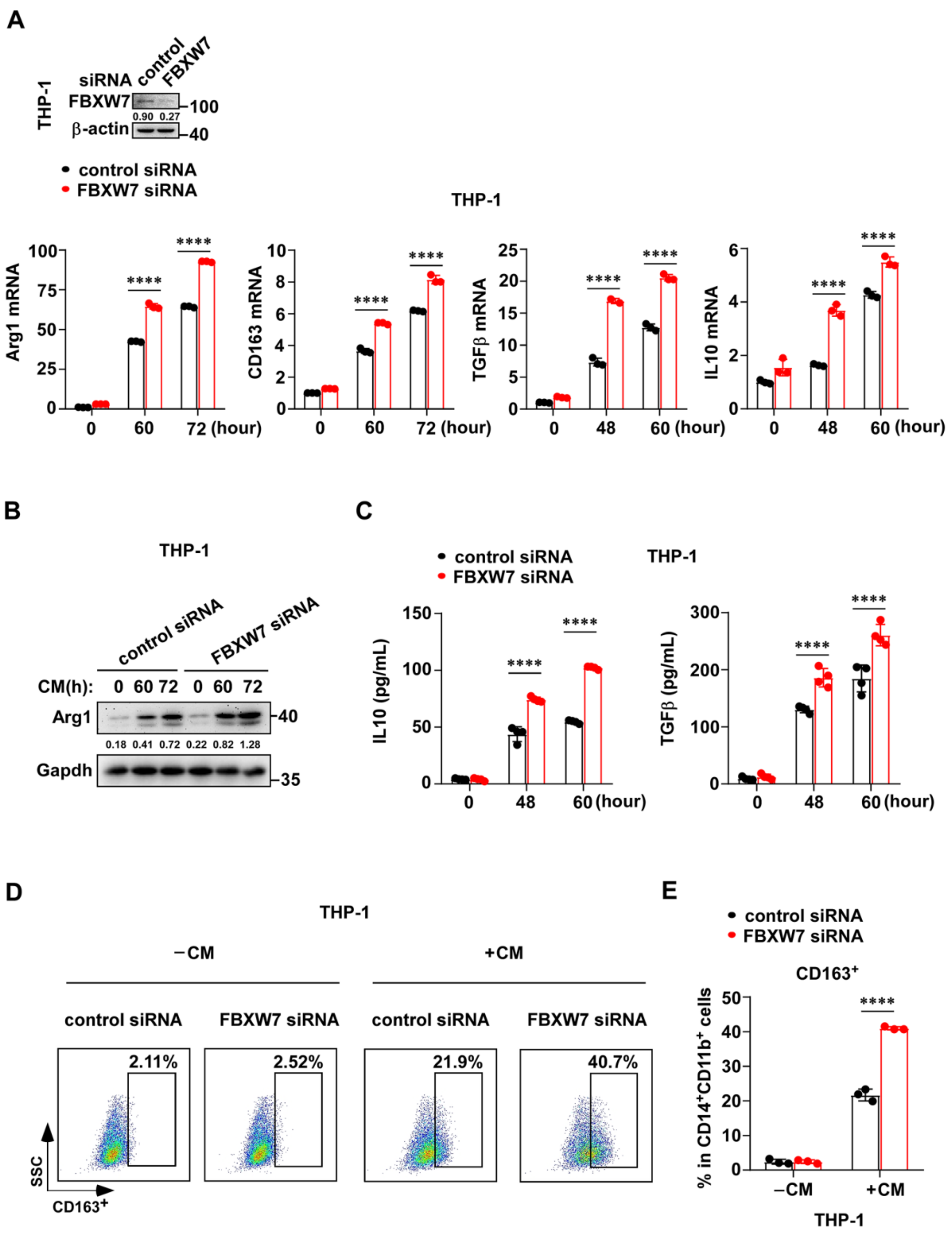

Supplementary Figure 4. FBXW7 knockdown in THP-1 promotes A549 supernatant-induced M2 macrophage polarization. (**A**) THP-1 cells were differentiated into macrophages in the presence of PMA for 48 hours and transfected with FBXW7 siRNA. The silenced and unsilenced THP-1 stimulated with conditioned medium containing A549 cells cultured supernatant. The protein expression of FBXW7 in THP-1 was examined by immunoblotting and the mRNA expression of *Arg1, CD163, TGFβ,* and *IL10* was detected by qRT-PCR. (**B**) The protein expression of Arg1 in two groups was examined by immunoblotting. (**C**) The protein levels of IL10 and TGFβ in the supernatant of wild-type and FBXW7-knockdown THP-1 that co-cultured with A549 cells for the indicated time were measured by ELISA kits. (**D**, **E**) Flow cytometry analysis (**D**) and statistical analysis (**E**) of the percentage of M2 macrophages ($CD163^+$) in wild-type and FBXW7-knockdown THP-1 after conditioned medium stimulation for 72 h (n = 3 per group). Data are shown as the mean ± SD and are representative of three independent experiments. *****P* < 0.0001; (two-way ANOVA (**A**, **C**, **E**)).

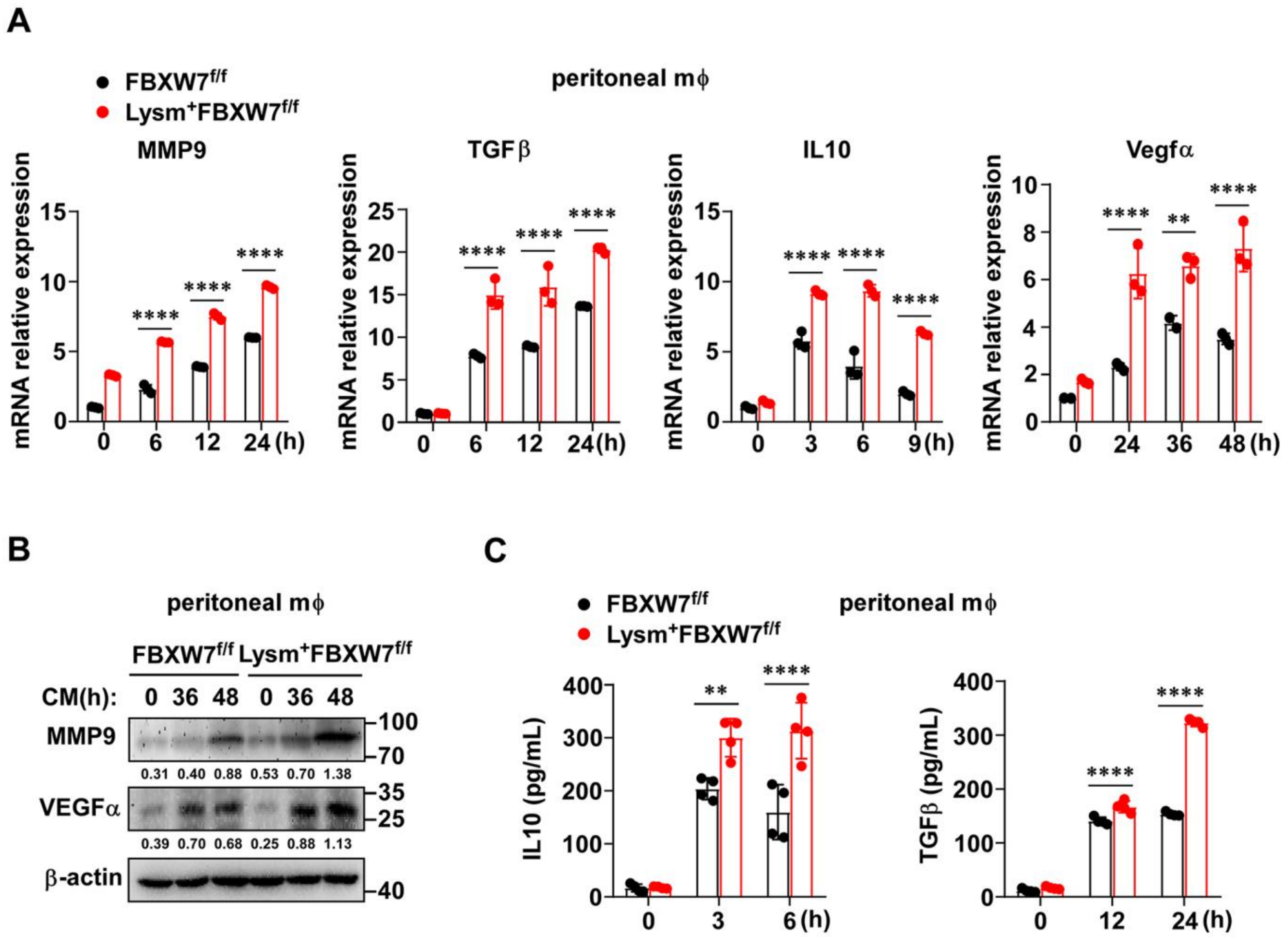

Supplementary Figure 5. FBXW7 knockout promotes the expression of pro-tumoral factors in peritoneal macrophages. (**A**) Peritoneal macrophages from FBXW7$^{f/f}$ and Lysm^{+}FBXW7$^{f/f}$ mice were stimulated with the conditioned medium, and the mRNA expression of *MMP9, IL-10, TGFβ,* and *VEGFα* was examined by qRT-PCR. (**B**) The protein expression of MMP9 and VEGFα in peritoneal macrophages incubated with the conditioned medium were detected by immunoblotting. (**C**) The protein levels of IL10 and TGFβ in the supernatant of peritoneal macrophages that co-cultured with LLCs for the indicated time was measured by ELISA kits. Data are shown as the mean ± SD and are representative of three independent experiments. $^{**}P < 0.01$; $^{****}P < 0.0001$ (two-way ANOVA (**A**, **C**)).

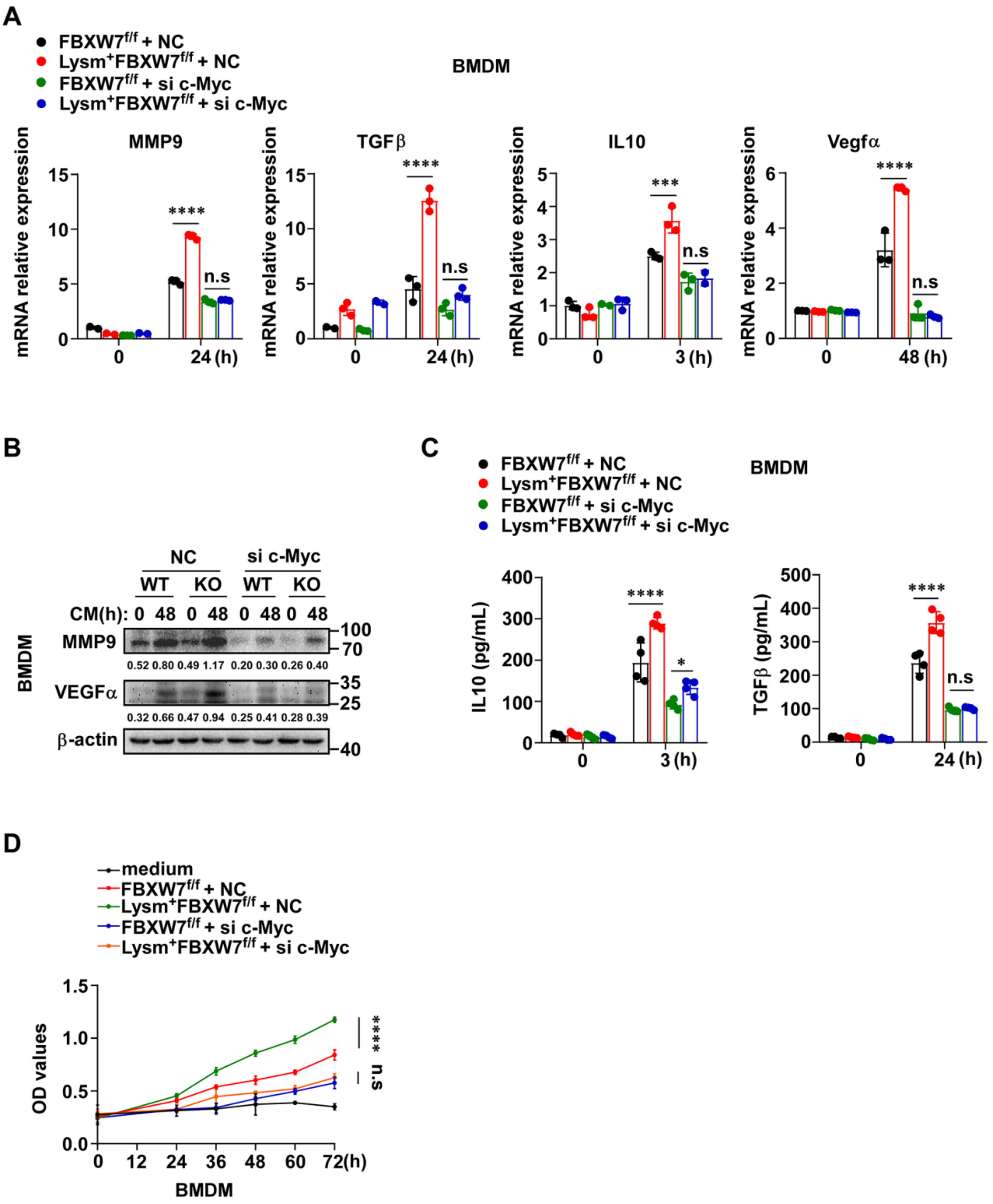

Supplementary Figure 6. The effect of FBXW7 on M2 macrophage expression of pro-tumoral factors is dependent on c-Myc. (**A**) qRT-PCR analysis of *MMP9*, *IL-10, TGFβ,* and *VEGFα* mRNA expression in BMDMs from FBXW7$^{f/f}$ and Lysm^{+}FBXW7$^{f/f}$ mice transfected with or without c-Myc siRNA and stimulated with conditioned medium. (**B**) Immunoblotting analysis of MMP9 and VEGFα expression in primary macrophages from FBXW7$^{f/f}$ and Lysm^{+}FBXW7$^{f/f}$ mice transfected with or without c-Myc siRNA and stimulated with conditioned medium. (**C**) The protein levels of IL10 and TGFβ in the supernatant of four groups were measured by ELISA kits. (**D**) LLCs were cultured in serum-free RPMI-1640, supernatant from IL-4-induced wild-type or FBXW7-knockout macrophages, supernatant from IL-4-induced wild-type or FBXW7-knockout macrophages transfected with c-Myc siRNA. The proliferation of LLCs in five groups was measured by the MTT assay. Data are shown as the mean ± SD and are representative of three independent experiments. *$P < 0.05$; ***$P < 0.001$; ****$P < 0.0001$; n.s, no significance (two-way ANOVA (**A**, **C**, **D**)).
