# Supplementary material for: E3 ligase FBXW7 restricts M2-like tumor-associated macrophage polarization by targeting c-Myc
Source: Aging (Albany NY). 2020 Dec 1;12(23):24394–423. doi: 10.18632/aging.202293 (PMC7762499; doi:10.18632/aging.202293)
Supplement: Supplementary Tables [file aging-12-202293-s002.pdf]

SUPPLEMENTARY TABLES

Supplementary Table 1. A list of antibodies and reagents used in the study.

REAGENTS	SOURCE	Cat#
**Antibodies**		
β-Actin (Mouse mAb)	Protein Tech	60008-1-Ig
GAPDH (Mouse mAb)	Protein Tech	60004-1-Ig
FBXW7 (Rabbit mAb)	Abcam	12992
Phospho-c-Myc T58 (Rabbit mAb)	Abcam	185655
Ym1 (Rabbit mAb)	Abcam	93034
c-Myc (Rabbit mAb)	Cell Signaling Technology	9402
Phospho-ERK (Rabbit mAb)	Cell Signaling Technology	4370
ERK (Rabbit mAb)	Cell Signaling Technology	4695
Phospho-JNK (Rabbit mAb)	Cell Signaling Technology	9251
JNK (Rabbit mAb)	Cell Signaling Technology	9252
Phospho-AKT S473 (Rabbit mAb)	Cell Signaling Technology	4060
Phospho-AKT T308 (Rabbit mAb)	Cell Signaling Technology	13038
AKT (Rabbit mAb)	Cell Signaling Technology	9272
Phospho-STAT6 (Rabbit mAb)	Cell Signaling Technology	56554
Arginase-1 (Rabbit mAb)	Cell Signaling Technology	93668
K48-linkage specific polyubiquitin (Rabbit pAb)	Cell Signaling Technology	8081
STAT6 (Rabbit mAb)	Themo Fisher Scientific	MA5-15659
MMP9	Santa Cruz Biotechnology	sc-393859
VEGF	Santa Cruz Biotechnology	sc-7269
FC: CD45	biolegend	103116
FC: CD11c	biolegend	117310
FC: CD11b	biolegend	101206
FC: F4/80	biolegend	123131
FC: CD206	biolegend	141720
FC: CD3	biolegend	100236
FC: CD4	biolegend	100434
FC: B220	biolegend	103206
FC: Ly6G	biolegend	127639
FC: Ly6C	biolegend	128014
FC: CD163	biolegend	333613
FC; CD14	biolegend	367143
FC: CD11b	biolegend	301309
FC: MHC II	eBioscience	12-5321-82
FC: CD8	eBioscience	12-0081-82
**Chemicals, Peptides, and Recombinant Proteins**		
Recombinant Murine IL4	PeproTech	214-14

Cycloheximide (CHX)	Sigma Aldrich	C4859
MG132	Sigma Aldrich	M8699
Liberase TM	Roche	345474
DnaseI	Themo Fisher Scientific	EN0521
PMA	Sigma Chemical	P1585
Mouse IL10 uncoated ELISA	Invitrogen	88-7105
Human IL10 uncoated ELISA	Invitrogen	88-7106
Human/Mouse TGF beta1 uncoated ELISA	Invitrogen	88-8350
**Experimental Models: Cell Lines**		
Lewis lung carcinoma cells (LLCs)	The Cell Bank of The Chinese Academy of Science,Shanghai,China	TCM 7
A549	The Cell Bank of The Chinese Academy of Science,Shanghai,China	SCSP-503
THP-1	The Cell Bank of The Chinese Academy of Science,Shanghai,China	SCSP-567
**Experimental Models: Organisms/Strains**		
C57BL/6J mice	Model Animal Research Center of Nanjing University	N000013
FBXW7$^{f/f}$ C57BL/6J mice	the Jackson Laboratary	017563
Lysm-Cre C57BL/6J mice	Prof. Ximei Wu of Zhejiang University.	not available
**Oligonucleotides**		
siRNA targeting sequence:c-Myc#1 CCGTACAGCCCTATTTCAT	this paper	not available
siRNA targeting sequence:FBXW7 CCAGAGAAATTGCTTGCTT	this paper	not available
**Software and Algorithms**		
FlowJO	FlowJO	https://www.flowjo.com/
imageJ	imageJ	https://www.imagej.net/
Graphpad Prism 8	Graphpad software	http://www.graphpad.com

Supplementary Table 2. Primers for RT-PCR.

Gene (mouse)	Forward (5′-3′)	Reverse (5′-3′)
β-actin	AGTGTGACGTTGACATCCGT	GCAGCTCAGTAACAGTCCGC
Arginase-1	CTCCAAGCCAAAGTCCTTAGAG	AGGAGCTGTCATTAGGGACATC
Fizz1	CCAATCCAGCTAACTATCCCTCC	CCAGTCAACGAGTAAGCACAG
Ym1	CAGGTCTGGCAATTCTTCTGAA	GTCTTGCTCATGTGTGTAAGTGA
FBXW7	GTGATAGAGCCCCAGTTCCA	CCTCAGCCAAAATTCTCCAG
MMP9	CTGGACAGCCAGACACTAAAG	CTCGCGGCAAGTCTTCAGAG
IL-10	TGGCCCAGAAATCAAGGAGC	CAGCAGACTCAATACACACT
VEGFα	GGAGATCCTTCGAGGAGCACTT	GGCGATTTAGCAGCAGATATAAGAA
TGFβ	GAAGGCAGAGTTCAGGGTCTT	GGTTCCTGTCTTTGTGGTGAA
c-Myc	ATGCCCCTCAACGTGAACTTC	CGCAACATAGGATGGAGAGCA

Supplementary Table 3. Primers for RT-PCR.

Gene (human)	Forward (5′-3′)	Reverse (5′-3′)
β-actin	CTCCATCCTGGCCTCGCTGT	GCTGCTACCTCCACCGTTCC
Arginase-1	TGGACAGACTAGGAATTGGCA	CCAGTCCGTCAACATCAAAACT
CD163	GACGCATTTGGATGGATCATGT	CCCACCGTCCTTGGAATTTGA
IL10	TCAAGGCGCATGTGAACTCC	GATGTCAAACTCACTCATGGCT
TGFβ	CTAATGGTGGAAACCCACAACG	TATCGCCAGGAATTGTTGCTG
